# Molecular Dynamics Simulation-assisted Ionic Liquid Screening for Deep Coverage Proteome Analysis

**DOI:** 10.1074/mcp.TIR119.001827

**Published:** 2020-11-25

**Authors:** Fei Fang, Qun Zhao, Huiying Chu, Mingwei Liu, Baofeng Zhao, Zhen Liang, Lihua Zhang, Guohui Li, Liming Wang, Jun Qin, Yukui Zhang

**Affiliations:** 1 CAS Key Laboratory of Separation Science for Analytical Chemistry, Dalian Institute of Chemical Physics, Chinese Academy of Science, National Chromatographic Research and Analysis Center, Dalian, China; 2 Laboratory of Molecular Modeling and Design, State Key Laboratory of Molecular Reaction Dynamics, Dalian Institute of Chemical Physics, Chinese Academy of Science, Dalian, China; 3 State Key Laboratory of Proteomics, Beijing Proteome Research Center, Beijing Institute of Radiation Medicine; National Center for Protein Sciences, Beijing, China; 4 Division of Hepatobiliary and Pancreatic Surgery, Department of General Surgery, The Second Affiliated Hospital of Dalian Medical University, Dalian, China; 5 Alkek Center for Molecular Discovery, Verna and Marrs McLean Department of Biochemistry and Molecular Biology, Department of Molecular and Cellular Biology, Baylor College of Medicine, Houston, Texas, USA

**Keywords:** Molecular dynamics simulation, ionic liquid, deep coverage proteome analysis, molecular dynamics*, quantification, omics, mass spectrometry, liver cancer, HPLC

## Abstract

In-depth coverage of proteomic analysis could enhance our understanding to the mechanism of the protein functions. Unfortunately, many highly hydrophobic proteins and low-abundance proteins, which play critical roles in signaling networks, are easily lost during sample preparation, mainly attributed to the fact that very few extractants can simultaneously satisfy the requirements on strong solubilizing ability to membrane proteins and good enzyme compatibility. Thus, it is urgent to screen out ideal extractant from the huge compound libraries in a fast and effective way. Herein, by investigating the interior mechanism of extractants on the membrane proteins solubilization and trypsin compatibility, a molecular dynamics simulation system was established as complement to the experimental procedure to narrow down the scope of candidates for proteomics analysis. The simulation data shows that the van der Waals interaction between cation group of ionic liquid and membrane protein is the dominant factor in determining protein solubilization. In combination with the experimental data, 1-dodecyl-3-methylimidazolium chloride (C12Im-Cl) is on the shortlist for the suitable candidates from comprehensive aspects. Inspired by the advantages of C12Im-Cl, an ionic liquid-based filter-aided sample preparation (*i*-FASP) method was developed. Using this strategy, over 3,300 proteins were confidently identified from 10^3^ HeLa cells (∼100 ng proteins) in a single run, an improvement of 53% over the conventional FASP method. Then the *i*-FASP method was further successfully applied to the label-free relative quantitation of human liver cancer and para-carcinoma tissues with obviously improved accuracy, reproducibility and coverage than the commonly used urea-based FASP method. The above results demonstrated that the *i*-FASP method could be performed as a versatile tool for the in-depth coverage proteomic analysis of biological samples.

## Teaser

Molecular dynamics simulation was established to elucidate the underlying effects of extractants on protein solubilization and bioactivity. In combination with experimental procedure, 1-dodecyl-3-methylimidazolium chloride was found to be the suitable candidate, and an ionic liquid-based filter-aided sample preparation (*i*-FASP) method was developed. Using this strategy, 3,300 proteins were identified from 1,000 HeLa cells in a single run, an improvement of 53% over FASP method. The *i*-FASP method further showed improved accuracy, reproducibility, and coverage than FASP method in label-free quantitative analysis.

## Graphical Abstract

**Figure:**
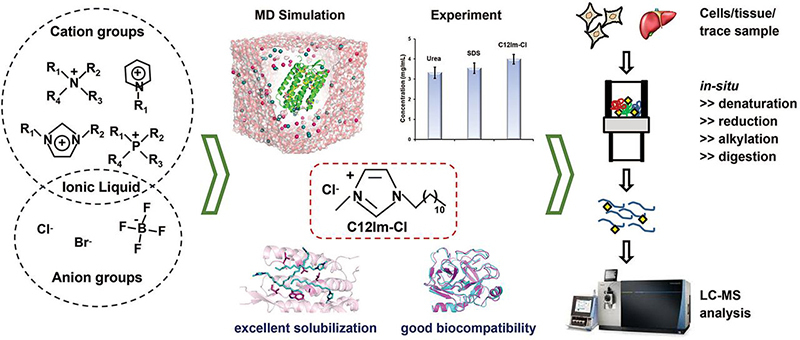

**Highlights**

• Mechanistic insights into ionic liquids and proteins at molecular level.
• Extractants prescreen for proteome analysis with MD simulation system.
• A loss-less sample preparation method developed for in-depth proteome profiling.
• Over 3,300 proteins were confidently identified from 1,000 HeLa cells in a 1 h run.
• Label-free quantitative proteome analysis of human liver cancer tissues.

With the rapid development of mass spectrometers and techniques, over 16,500 proteins encoded by genes have been elucidated with reliable experimental evidence (1, 2, 3, 4). However, many highly hydrophobic and low-abundance proteins are still easily lost during sample preparation, which impedes in-depth proteome analysis (5, 6, 7). Therefore, the development of sample preparation methods that are unbiased to such proteins is of great significance to achieve the comprehensive proteome analysis with deep coverage.

As the prerequisite and indispensable steps in 'shotgun' proteomics analysis, proteins solubilization and digestion were needed to be both high efficiency and good compatibility. Detergents, such as sodium dodecyl sulfonate (SDS), are commonly used for protein extraction because of their strong solubilizing ability (8). To avoid interference in the subsequent tryptic digestion and liquid chromatography coupled to tandem MS (LC–MS/MS) analysis, a filter-aided sample preparation (FASP) method had been developed and successfully applied to the study of various biological issues (9, 10, 11, 12). In this strategy, SDS could be removed by a multistep washing procedure involving urea and ammonium bicarbonate (NH_4_HCO_3_) buffers in the filter, which is not only time consuming but also result in sample loss (13). Additionally, it was found that the dissolving capability of SDS to proteins with extremely high hydrophobicity is still limited.

Recently, to realize the in-depth proteome profiling of low-nanogram samples, Dou *et al.* (14) developed a microfluidic nanodroplet-based sample processing platform. With this device, the biological samples containing sub-nanogram amounts of protein could be efficiently converted into ready-to-analyze peptides. However, this work is highly professional and special equipment is needed. Furthermore, the commonly used detergents with strong solubilizing ability could not be applied in their device considering the poor compatibility of these detergents with proteases.

In our previous studies, several ionic liquids (ILs) were found to have great performance for membrane proteins analysis (15, 16, 17, 18, 19, 20), showing the potential of this underestimated reagents to be extractant candidates. However, testing the limited number of the commercially available ILs with experimental tools is not enough for extractant screening, whereas it's unrealistic to synthesize and purify all potential ILs. In the present work, without worrying about much extra time and money for those unavailable extractants, a molecular dynamics simulation-based system, with which the interaction force of any ionic liquids and protein could be calculated, was established. This system could help to narrow down the candidate screening pool of ionic liquids, as well as elucidate the interior mechanism of extractant on membrane proteins solubilization and trypsin compatibility, which is difficult to be solved by experimental method alone. The simulation result showed that the methylimidazolium-based ILs containing alkyl chain length longer than C12 (CnIm-Cl, n = 12, 14, 16 …) having stronger interaction force with membrane proteins than other ILs as well as the commonly used extractant, and the van der Waals interaction between cation group of ionic liquid and membrane protein played the dominant role in determining protein solubilization. In combination with the experimental result (19), 1-dodecyl-3-methylimidazolium chloride (C12Im-Cl) was screened out as the ideal extractant. Moreover, we presented a robust and easy-to-use sample preparation method, ionic liquid-based FASP (*i*-FASP), and successfully applied our method in the qualitative analysis of trace HeLa cells and the quantitative analysis of human liver cancer and para-carcinoma tissues. The experimental results demonstrated the high feasibility by using the established simulation system to design the best extractant in various proteomics fields, and the *i*-FASP strategy could provide high accuracy, precision and throughput for the deep-coverage proteome analysis, especially for those highly hydrophobic and low-abundance proteins.

## EXPERIMENTAL PROCEDURES

##### Reagents and Materials

The ionic liquids, 1-butyl-3-methylimidazolium thiocyanate (C4Im-SCN) and 1-dodecyl-3-methylimidazolium chloride (C12Im-Cl), were obtained from Shanghai Cheng Jie Chemical (Shanghai, China). Sodium dodecyl sulfonate (SDS), bovine serum albumin (BSA), tris(hydroxymethyl)aminomethane (Tris), tris (2-carboxyethyl) phosphine (TCEP), iodoacetamide (IAA), ethylene diamine tetraacetic acid (EDTA), ethylene glycol tetraacetic acid (EGTA), ammonium bicarbonate (NH_4_HCO_3_) and protease inhibitor mixture were purchased from Sigma-Aldrich. Urea was bought from Fluka (Buchs, Germany), and sequencing-grade modified trypsin was from Promega. Acetonitrile (ACN) and methanol were purchased from Merck (Darmstadt, Germany). The Microcon filtration device with a relative molecular mass cut-off of 10 kDa was from Sartorius AG (Goettingen, Germany). Deionized water was purified using a Milli-Q system from Millipore. Other chemicals were of analytical grade. Durashell C18 particles (5 μm, 150 Å pore) were obtained from Bona-Agela Technologies (Tianjin, China). Reprosil-Pur C18-AQ particles (3 μm, 120 Å pore) were obtained from Dr. Maisch GmbH (Ammerbuch-Entringen, Germany). Fused-silica capillaries (100 μm i.d./360 μm o.d.) were purchased from Sino Sumtech (Handan, Hebei, China).

##### Solubility Measurement for Bacteriorhodopsin

Equal aliquots of bacteriorhodopsin (100 μg) were solubilized in 25 μL of each IL at 1% (w/v), including C4Im-SCN and C12Im-Cl in 50 mm NH_4_HCO_3_ buffer, as well as C4Im-SCN mixed with 0.5 M NaOH. The samples were respectively sonicated for 10 min in a water bath at room temperature. Afterward, the samples were centrifuged to remove insoluble materials, and the supernatants were collected. The samples were then quantified by the BCA protein assay kit (Bio-Rad) at 562 nm with BSA as the standard protein. All the measurements were repeated three times in parallel.

##### Cell Culture

HeLa cells were cultured in MEM-containing 10% fetal bovine serum (v/v) and maintained in a humidified atmosphere of 95% air and 5% CO_2_ at 37 °C. The adherent cell layer was washed with PBS and then trypsinized with 0.05% trypsin-EDTA solution for 5 min at 37 °C. Then, the cells were centrifuged at 250 × *g* for 5 min to remove trypsin, followed by washing with PBS for three times.

##### Optimization of Extraction Buffer

For protein extraction, frozen aliquots of 1 × 10^6^ HeLa cells were lysed respectively using 100 μl 0.1 M Tris buffer (pH 7.6) with 1%, 5%, 10 and 15% C12Im-Cl (w/v) in triplicate, to optimize the ionic liquid concentration. For comparison, 100 μl 8 M urea in 0.1 M Tris buffer (pH 8.5) and 4% SDS in 0.1 M Tris buffer (pH 7.6) were used for protein extraction and solubilization, respectively.

For the above different detergents, samples were respectively incubated at 95 °C for 3 min except that for urea, incubated at room temperature for 30 min. Each cell suspension was sonicated on ice for 20 s (pulse on time 5 s, pulse off time 15 s). Subsequently, the cell debris was removed by centrifugation at 16,000 × *g* at 4 °C for 5 min. Finally, BCA protein assay kits were used to measure the protein concentration of each supernatant.

##### Computational Details and Models

To perform molecular dynamics (MD) simulation, the bacteriorhodopsin (PDB code 1M0K (21)) was used to measure the interaction energies of the membrane protein with different chemical reagents, and the protein trypsin (PDB code 4AN7 (22)) was used to test the enzyme activity in different extractants. All molecular dynamics studies were carried out using the GROMACS 5.0.4 software package (23, 24). The all-atom force field expanded by Lopes *et al.* based on the OPLS-AA frame work was applied to CnIm-Cl, CnIm-Br, CnIm-BF_4_, NH_2_-CnIm-Cl, OH-CnIm-Cl, CH_2_=CH-CnIm-Cl, CN-CnIm-Cl (*n* = 2, 4, 6, 8, 10, 12, 14, 16) (25), whereas the GROMOS 53a6 force field parameters for SDS were adapted from the work of Tang *et al.* (26). The OPLS-AA parameters of methyltributylmethylammonium chloride, 1-dodecyl-3-methyl-pyridinium chloride, and methyltributylmethylphosphonium chloride were obtained from GMXTOP web server (https://Jerkwin.github.io/prog/gmxtop.html). Bacteriorhodopsin was respectively inserted in an 8.5 nm×8.5 nm×11 nm simulation box filled with 1% ionic liquid and 1% SDS (w/v). The systems contained 14 SDS molecules and 11 ionic liquid with TIP3P waters, respectively. The trypsin was inserted in an 8 nm×8 nm×8 nm cubic simulation box filled with two different systems respectively containing 11 SDS molecules and 10 ionic liquid molecules with TIP3P waters. These systems were first constructed using the Packmol package (27).

A periodic boundary condition was applied in all directions of the simulation box. The energy minimization without constraining any solvent atoms was followed by the minimization of the whole system over a few thousand steps to remove conflicting contacts. Subsequently, equilibrations of the systems were performed with gradual release of the position restraints on the protein (1 ns). Finally, for each system, MD simulations were carried out for 50 ns. All the simulations were performed under constant pressure (NPT) conditions. The linear constraint solver (LINCS) method (28) was used to constrain the bonds associated with H atoms, and the integration time step was set to 2 fs. Electrostatic interactions were calculated using the particle-mesh Ewald (PME) algorithm (29, 30). The cut-off distance of the neighbor searching was set to 1.2 nm. To maintain a constant temperature of 300 K, the Nose-Hoover thermostat (31) was applied with a coupling time of 0.1 ps. Therefore, when the NPT conditions were used, the Parinello-Rahman pressure coupling method, which works in conjunction with the Langevin thermostat, was applied at 1.0 bar.

##### Sample Preparation by i-FASP

**Fig. 2:**
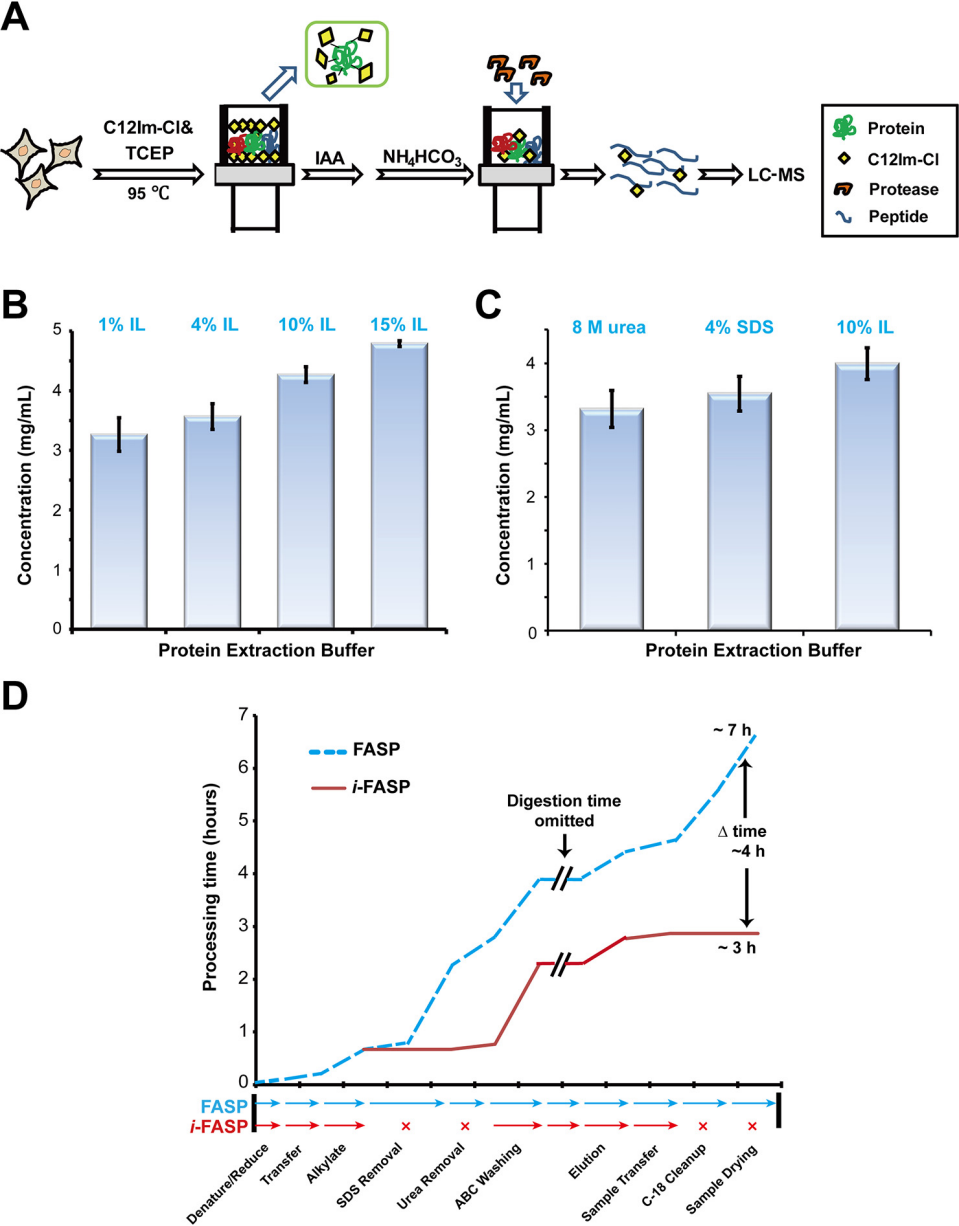
**Evaluation on performance of *i*-FASP method.***A*, Flowchart of the *i*-FASP method. *B*, The effects of the C12Im-Cl concentration on the protein extraction. *C*, A comparison of the efficiency on protein extraction using three extractants. *D*, A comparison of the sample preparation times for the *i*-FASP (*solid line*) and FASP (dash line) methods.

The protocol for the sample preparation of cells by *i*-FASP (10^6^ cells/experiment) was shown in Fig. 2*A*. For the HeLa cells, an aliquot of 10^6^ cells were solubilized by adding 0.2 ml of lysis buffer (10% C12Im-Cl and 100 mm TCEP dissolved in 100 mm Tris, pH 7.6, 1% protease inhibitor mixture (v/v)) followed by incubation at 95 °C for 3 min and sonication in a water-ice bath for further solubilization. The cell debris was removed by centrifugation at 16,000 × *g* for 5 min, and 40 μl of the clarified protein extract was transferred to a 10 kDa filter. After centrifugation on the filter at 14,000 × *g* at 20 °C for 15 min, the extracted proteins were retained, diluted with 200 μl of 50 mm NH_4_HCO_3_ and centrifuged again at 14,000 × *g* at 20 °C for 15 min. Subsequently, 200 μl of 50 mm IAA dissolved in 50 mm NH_4_HCO_3_ buffer was added, and the samples were incubated in darkness for 20 min. After the filters were washed three times with 200 μl of 50 mm NH_4_HCO_3_, 100 μl of 10 mm NH_4_HCO_3_ containing modified trypsin (5 μg) was added, and the sample was digested for overnight at 37 °C. Finally, the tryptic peptides were collected by centrifugation, and 100 μl of water was added to elute the peptide-rich solution.

The detailed information for the sample procession of 1000 HeLa cells and human liver tissue with *i*-FASP method was included in Supporting Information.

##### Sample Preparation by FASP

The cells (10^6^ cells/experiment) was prepared using the FASP method following the procedures described previously with minor modifications (9). Briefly, each aliquot of 1 × 10^6^ cells was solubilized by 0.2 ml of lysis buffer (4% SDS and 100 mm TCEP in 100 mm Tris, pH 7.6, 1% protease inhibitor mixture (v/v)). Then, the samples were incubated at 95 °C for 3 min with ultrasonication for 20 s (pulse on time 5 s, pulse off time 15 s) to reduce the viscosity of the lysate and sonicated in a water-ice bath for further solubilization. The cell debris was removed by centrifugation at 16,000 × *g* for 5 min, and 40 μl of the clarified protein extract was transferred to a 10 kDa filter. After centrifugation at 14,000 × *g* at 20 °C for 15 min, the protein sample was retained on the filter. The concentrate was diluted in the device with 200 μl of UA buffer (8 M urea dissolved in 50 mm NH_4_HCO_3_) and centrifuged at 14,000 × *g* at 20 °C for 15 min. Subsequently, 200 μl of 50 mm IAA dissolved in UA buffer was added to the filter, and the samples were incubated in darkness for 20 min. Next, the filters were washed three times with 200 μl of UA buffer and 200 μl 50 mm NH_4_HCO_3_, respectively. After that, 5 μg of trypsin in 100 μl of 10 mm NH_4_HCO_3_ was added to the sample, and the samples were then digested overnight at 37 °C. Finally, the tryptic peptides were collected by centrifugation, and 100 μl of water was added to elute the peptide-rich solution.

The detailed information for the sample processing of 1000 HeLa cells and human liver tissue with FASP method is included in Supporting Information.

##### Sample Preparation by in-Solution Digestion Method

The cells (10^6^ cells/experiment) was prepared using the in-solution digestion method following the procedures described previously (15). In brief, three aliquots containing 1 × 10^6^ cells were solubilized in 4% C12Im-Cl (w/v, in 50 mm NH_4_HCO_3_, pH 8.0). Each cell suspension was sonicated on ice for 60 s (pulse on time 10 s, pulse off time 10 s). Subsequently, the cell debris was removed by centrifugation at 16,000 × *g* at 4 °C for 5 min, and the supernatant were maintained at 90 °C for 20 min for thermal denaturation. Subsequently, the samples were cooled, followed by reduction in 10 mm TCEP at 56 °C for 1 h. Afterward, the cysteines were alkylated in darkness in 25 mm IAA for 30 min at room temperature. The tryptic digestion was diluted by addition of 50 mm NH_4_HCO_3_ to 5 times and performed with a trypsin/protein ratio (m/m) of 1:25 at 37 °C for 12 h. Tryptic digests were precipitated with bis(trifluoromethane)sulfonimide lithium salt for C12Im-Cl removal, followed by desalting with a homemade C18 trap column.

##### Nano RPLC-ESI-MS/MS Analysis

The detailed information was included in Supporting Information.

##### Database Searching

The nano-RPLC-ESI-MS/MS raw files obtained with the Q-Exactive mass spectrometer were searched against the Human UniProtKB database (release 2019_8, 20,434 protein entries) using Proteome Discoverer 2.2.0.388 and MaxQuant v1.6.7.0. Peptides were searched using fully tryptic cleavage constraints, and up to two cleavage sites were allowed for tryptic digestion. Cysteine carbamidomethylation was set as a static modification, whereas N-terminal acetylation and methionine oxidation were set as variable modifications. Peptide identification was based on a search with an initial mass deviation of up to 6 ppm for the precursor ions and an allowed fragment mass deviation of 20 ppm. A false discovery rate (FDR) of 0.01 for both proteins and peptides were required to filter the results.

The nano-RPLC-ESI-MS/MS raw files obtained by the Orbitrap Fusion Lumos mass spectrometer were searched against the Human UniProtKB database (release 2016_7, 42,164 protein entries) with the Proteome Discoverer 2.1.0.81. Peptides were searched using fully tryptic cleavage constraints, and up to two cleavage sites were allowed for tryptic digestion. Cysteine carbamidomethylation was set as a static modification, whereas N-terminal acetylation and methionine oxidation were set as variable modifications. The mass tolerances for MS and MS/MS were set to 20 ppm and 0.5 Da, respectively.

For the identification quality control, the target-decoy based strategy was applied to keep both peptide and protein level false discovery rates (FDRs) lower than 1%. PepDistiller was used to calculate the probability value (q-value) that kept the FDR measured by the decoy hits lower than 1% for every peptide-spectrum matches (PSMs). The peptides with length longer than 6 amino acids and shorter than 60 amino acids were kept. The q-values of both target and decoy peptide sequences were dynamically increased until the corresponding protein FDR was less than 1% employing the parsimony principle. FDR estimation on the level of peptide spectrum matches was performed using the peptide validator node with filtering for 1% FDR (high confidence filter). For the fractionated sample, all the PSMs in all fractions were combined to achieve a more stringent quality control.

##### Experimental Design and Statistical Rationale

In each experiment, three biological replicates were compared. For the quantification analysis, a home-made program was used to carry out statistics. Briefly, the proteins quantified in triplicates were remained for subsequent analysis, and a two-tailed *t* test applied with correction for multiple testing (Benjamini-Hochberg). Volcano plots were constructed using the permutation-based approach of Tusher and coworkers (32), to implement an FDR of 0.01. All statistical analysis was performed using GraphPad software (Graphpad Prism, RRID:SCR_002798). Statistical significance between datasets was determined by performing two-tailed, Wilcoxin test. In statistical analysis, *p* > 0.05 is indicated as not significant (n.s.), whereas statistically significant values are indicated by asterisks as follows: ***p < 0.001, ****p < 0.0001.

##### Bioinformatics Analysis

The assignment of protein cellular localization and molecular function was according to the Gene Ontology (GO)-annotation, the UniProtKB database (http://www.uniprot.org/), and the Database for Annotation, Visualization and Integrated Discovery (DAVID) v6.8 (https://david.ncifcrf.gov/) (33, 34). TMHMM algorithm (http://www.cbs.dtu.dk/services/ TMHMM/) was used to predict transmembrane domains (TMDs) of identified proteins and the transmembrane peptides. The topology of protein was visualized with the Protter software (http://wlab.ethz.ch/protter/). The protein interaction network analysis was performed by STRING software v10.0 (http://www.string-db.org/). Disease network and regulator effect analysis were performed by using Ingenuity Pathway Analysis (IPA, Ingenuity Systems Inc).

## RESULTS

##### Theoretical Simulation of Ionic Liquid-Based Membrane Protein Solubilization and Trypsin Biocompatibility

To screen out an ideal extractant for in-depth proteome analysis from a myriad of chemical reagents, it's crucial to develop an efficient platform to narrow down the scope of candidates before experimental procedure. Molecular dynamics (MD) (35, 36), an overview system of the dynamic evolution, was employed to simulate the interaction between the small molecules and the proteins, as well as elucidate the interior mechanism of extractants on the membrane proteins solubilization and trypsin biocompatibility. As several ionic liquids (ILs) were found to have great performance in our previous proteomics research (15, 16, 17, 18, 19), various ILs fine-tuned by the independent selection of cation and anion groups were applied to the simulation system as a pool to prescreen out the extractant candidates.

First, an equation was needed to calculate the interaction energy between the small molecules and proteins. In simulations system, the forces between atoms and the potential energy of the system are defined by molecular mechanics biomolecular force fields, which are parameterized to fit quantum-mechanical calculations and experimental spectroscopic data. Parameterization involves definition of chemical bonding, atomic angles and dihedral angles, as well as determination of partial atomic charges for calculation of the electrostatic-interaction energies, identification of appropriate van der Waals atomic radii, etc (37). The detailed modeling and parameters extraction performed in our simulation system were illustrated in the Methods Section. When equilibrium is reached, the interaction energy between the protein and solvent is calculated based on the following equation in triplicate

$$\Delta E_{ele+vdW}=E_{ele+vdW}^{protein+solvent}-E_{ele+vdW}^{protein}-E_{ele+vdW}^{solvent}$$

where $E_{ele+vdW}^{protein+solvent}$ is the sum of the electrostatic and van der Waals interaction energy of protein dissolving in solvent, whereas $E_{ele+vdW}^{protein}$ and $E_{ele+vdW}^{solvent}$ respectively denote the energy of protein and the solvent. In this work, the systems of bacteriorhodopsin (BR), an integral membrane protein with 7 transmembrane helices, was selected as the model and dissolved in different ILs with four commonly used cation groups, including two branched ILs of methyltributylammonium chloride and methyltributylphosphonium chloride, as well as two linear ILs of 1-dodecyl-3-methylimidazolium chloride (C12Im-Cl) and 1-dodecyl-3-methylpyridinium chloride (C12Py-Cl), were under simulation. As shown in supplemental Table S1, the imidazolium and pyridinium-based ILs had the comparable interaction energy when binding with BR, with at least 15% increase obtained compared with that between the branched ILs and BR (856.85 *versus* 734.33 KJ/mol). Whereas the Cl^−^ contributed negligible portions to the total interaction force, the van der Waals energy between cation groups of ILs and BR accounted for a large proportion in determining its solubilizing ability to membrane protein.

Furthermore, various methylimidazolium chlorides with different lengths of alkyl side chains (CnIm-Cl, *n* = 2, 4, 6, 8, 10, 12, 14 and 16) were respectively modeled. The result illustrated that the total interaction energy between BR and CnIm-Cl was strengthened as the length of the alkyl side chain increased; however, it reached a plateau at the C12Im-Cl (supplemental Table S2). The rule was further proved by evaluating the CnIm-based ILs with other commonly used anion groups like Br^-^ and BF_4_^−^ (supplemental Table S3 and S4). The simulation data showed that there was no obvious difference among the interaction forces of BR and C12Im-based ILs with different anion groups. However, because the water solubility of the CnIm-Br and CnIm-BF_4_ became dramatically worse as the length of the alkyl side chain increased, the ILs containing Cl^−^ were preferred to be extractant candidates for proteome analysis.

The ILs bearing functional groups were also subjected to the simulation system. As shown in supplemental Table S5–S8, both the π − π conjugate interactions of cations with the unsaturated alkyl substituent (CH_2_=CH-C10Im-Cl and CN-C12Im-Cl) and the hydrogen bond interaction of the cations with (-NH_2_ and -OH) substituent contributed little to the ILs on the interaction energy. Interestingly, the rule of the total interaction energy between BR and CnIm-Cl was strengthened as the length of the alkyl side chain increased and reached a plateau when *n* = 12 still works for these specific ILs.

**Fig. 1:**
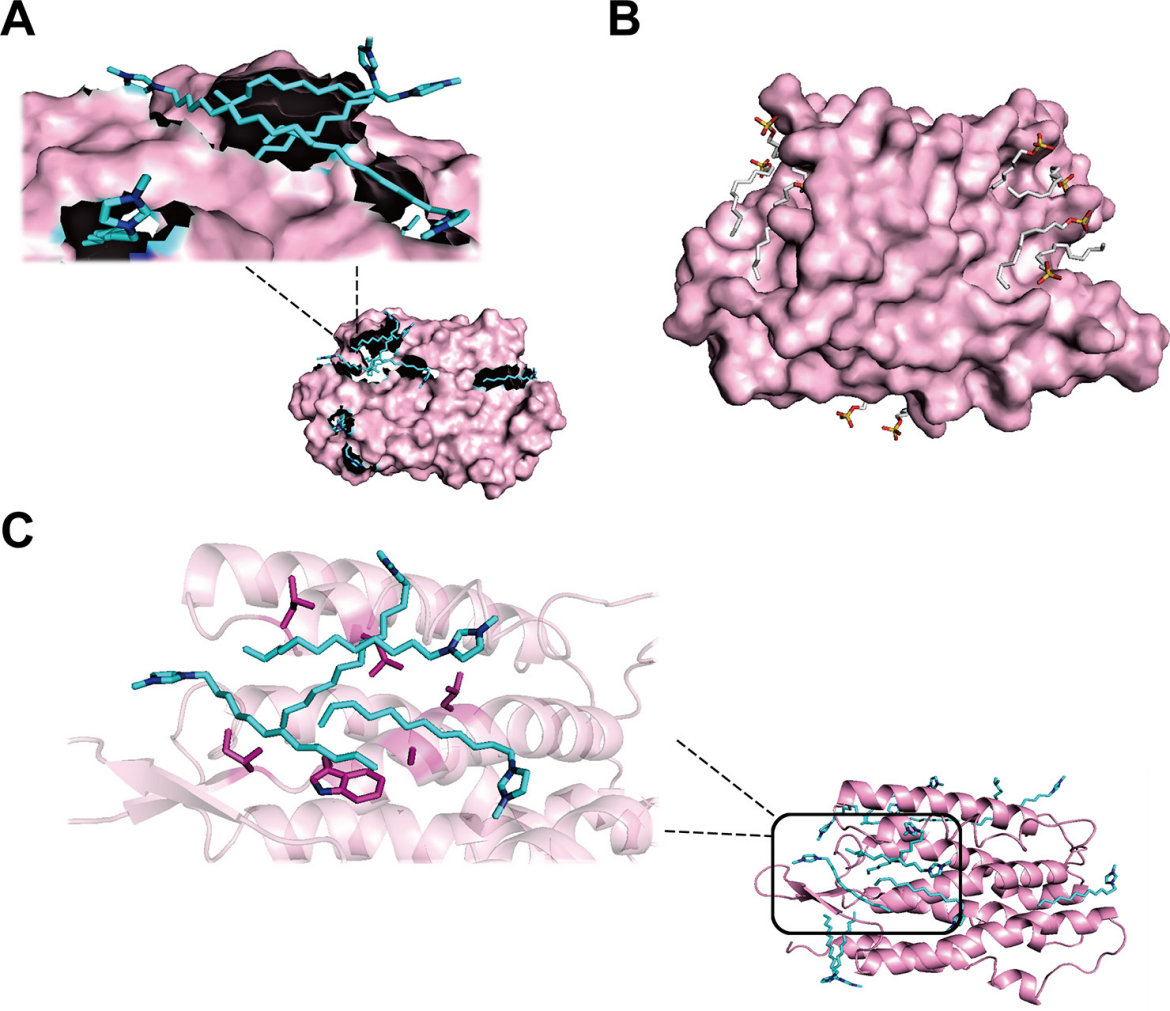
**Conformation of bacteriorhodopsin-extractants simulation system.** Snapshots of direct adsorption between the surface of bacteriorhodopsin and (*A*) cation group of C12Im-Cl or (*B*) anion group of SDS; and (*C*) the binding sites between the C12Im-Cl and the surface of bacteriorhodopsin. The bacteriorhodopsin is shown in violet, C12Im-Cl is shown in cyan, and SDS is shown in gray.

In combination with the above result and our previous experimental result (19), as well as the poor water solubility of the CnIm-Cl (n > 12), the C12Im-Cl was on the shortlist for the ideal extractants with strong solubilization capability to membrane proteins. Sodium dodecyl sulfate (SDS), which has been widely considered as one of the strongest detergents with high solubilizing ability, was taken as the reference for the simulation system. It was found that, compared with the tight interaction between C12Im-Cl and BR (Fig. 1*A*), SDS and BR were much farther apart (Fig. 1*B*), which was in accordance with our previous result that SDS provided a relatively weaker dissolving capacity than C12Im-Cl.

To further investigate the performance of the MD simulation for prescreen of protein extractant. The 1-butyl-3-methylimidazolium thiocyanate (C4Im-SCN), an ionic liquid was found that experimentally work to extract proteins very efficiently (38), was subjected to our MD simulation. The result showed that the interaction force between C4Im-SCN and BR was much weaker than that of C12Im-Cl (supplemental Table S9). We also performed experimental procedure to validate our simulation result. As shown in the supplemental Fig. S1, with bacteriorhodopsin as protein sample, the C4Im-SCN in 50 mm NH_4_HCO_3_ almost extracted nothing; even when it was mixed with the alkali solvent of 0.5 M NaOH, very few amounts of membrane protein were extracted. Oppositely, a large amount of membrane protein could be extracted and dissolved by C12Im-Cl.

To reveal the mechanism of the high efficiency of C12Im-Cl on membrane proteins solubilization, the weights of different interaction forces devoted to the total energy were compared. Notably, the van der Waals energy between [CnIm]^+^ and BR was the dominant factor in the total interaction energy for each IL (supplemental Table S2–S8). Furthermore, as shown in Fig. 1*A*, the conformation for the BR-C12Im-Cl system demonstrated that it was the alkyl chain, rather than the cation ring of C12Im-Cl, adsorbed onto the surface of BR, and the C12Im-Cl was mainly bound to the exposed hydrophobic amino acid residues of the membrane protein (Fig. 1*C*). Hence, it concluded that C12Im-Cl could efficiently solubilize membrane proteins because of strong hydrophobic interactions.

To evaluate the compatibility of extractants with trypsin activity, the conformations of trypsin dissolved in water, C12Im-Cl and SDS were simulated separately. First, to determine the overall stability of the developed system, the root-mean-square deviations (RMSD) from the starting structure were calculated for all Cα of trypsin in water, C12Im-Cl and SDS systems against simulation time (supplemental Fig. S2A). As shown in supplemental Fig. S2A, with the equilibrated protein structures obtained, trypsin dissolved in C12Im-Cl displayed the least motion in all the MD simulations; it rapidly equilibrated within ∼1 ns, and the RMSD remained constant throughout the rest of the simulation, with an average value of 1 Å, similar to that of trypsin dissolved in water. By contrast, the equilibration of trypsin in SDS took ∼3 ns, with the average RMSD of 1.6 Å. Similar to that observed in water (supplemental Fig. S2B), there was little change in the conformation of trypsin dissolved in C12Im-Cl compared with the native crystal structure (supplemental Fig. S2C). However, when trypsin was dissolved in SDS, parts of the twisted β-sheets became flexible, and the positions of α helices changed substantially (supplemental Fig. S2D). The phenomenon was also reflected in the more obvious fluctuation (shown as larger root-mean-square fluctuation value) in the positions of each amino acid of trypsin in SDS system (supplemental Fig. S2E). As the center of the active site of protease, the change occurred in the conformation of the catalytic triad would dramatically attenuate the enzyme activity of trypsin. As shown in supplemental Fig. S2F, unlike the obvious change observed in SDS system, the position and orientation of the catalytic triad in C12Im-Cl system were consistent with those of the native crystal structure, crucial for maintaining the enzyme activity of trypsin. The above results demonstrated the excellent trypsin biocompatibility of C12Im-Cl. This is the first time to elucidate the interior interaction mechanism between ILs and proteins at molecular level. As two independent events, the simulation result was in perfect accord with the experimental data, demonstrating that the molecular dynamics simulation system could be an important complement to experimental procedures for screening out the optimal extractant for proteome analysis.

##### Establishment of i-FASP Method

Based on the superiority of C12Im-Cl regarding membrane protein solubilization and trypsin biocompatibility, C12Im-Cl has been utilized in the analysis of membrane proteins from large amounts of samples in our previous work. However, the C12Im-Cl were removed by using strong cation exchange, inducing the sample loss, which limited its applications in the trace amount samples, as well as the in-depth coverage analysis of proteomics. With an entrenchment at every step, we established an ionic liquid-based filter-aided sample preparation (*i*-FASP) method to achieve the in-depth proteome analysis in this work (Fig. 2*A*). With 1 × 10^6^ HeLa cells as the sample, the lysis buffer containing C12Im-Cl was used to disrupt the cells and extract proteins at 95 °C, and we observed that the concentration of extracted protein increased with that of C12Im-Cl (Fig. 2*B*). C12Im-Cl with 10% (w/v) was chosen as the optimal system because it was more efficient than the 4% SDS and 8 M urea in proteins extraction (Fig. 2*C*). Subsequently, *in situ* alkylation, desalting and digestion were integrated on a filter with a cut-off molecular weight of 10 kDa. Unlike the conventional FASP method using SDS as the extractant, repeated washing steps were not needed to remove the detergent from the C12Im-Cl/trypsin solution because of their excellent compatibility. After on-filter digestion with trypsin in 10 mm NH_4_HCO_3_, an equal volume of water was used to elute the residual peptides without additional desalting steps for peptide purification. Therefore, the sample preparation time could be shortened by ca. 4h relative to FASP (Fig. 2*D*).

To further demonstrate the potential of *i*-FASP, we performed the qualitative proteome analysis of 1 × 10^6^ HeLa cells. First, the retention behavior of residual C12Im-Cl on LC–MS/MS was tested to evaluate the compatibility of C12Im-Cl with MS analysis. As shown in supplemental Fig. S3A, the residual C12Im-Cl was eluted until the mobile phase composed of 80% ACN (v/v), with no negative effects on the identification of peptides.

**Fig. 3:**
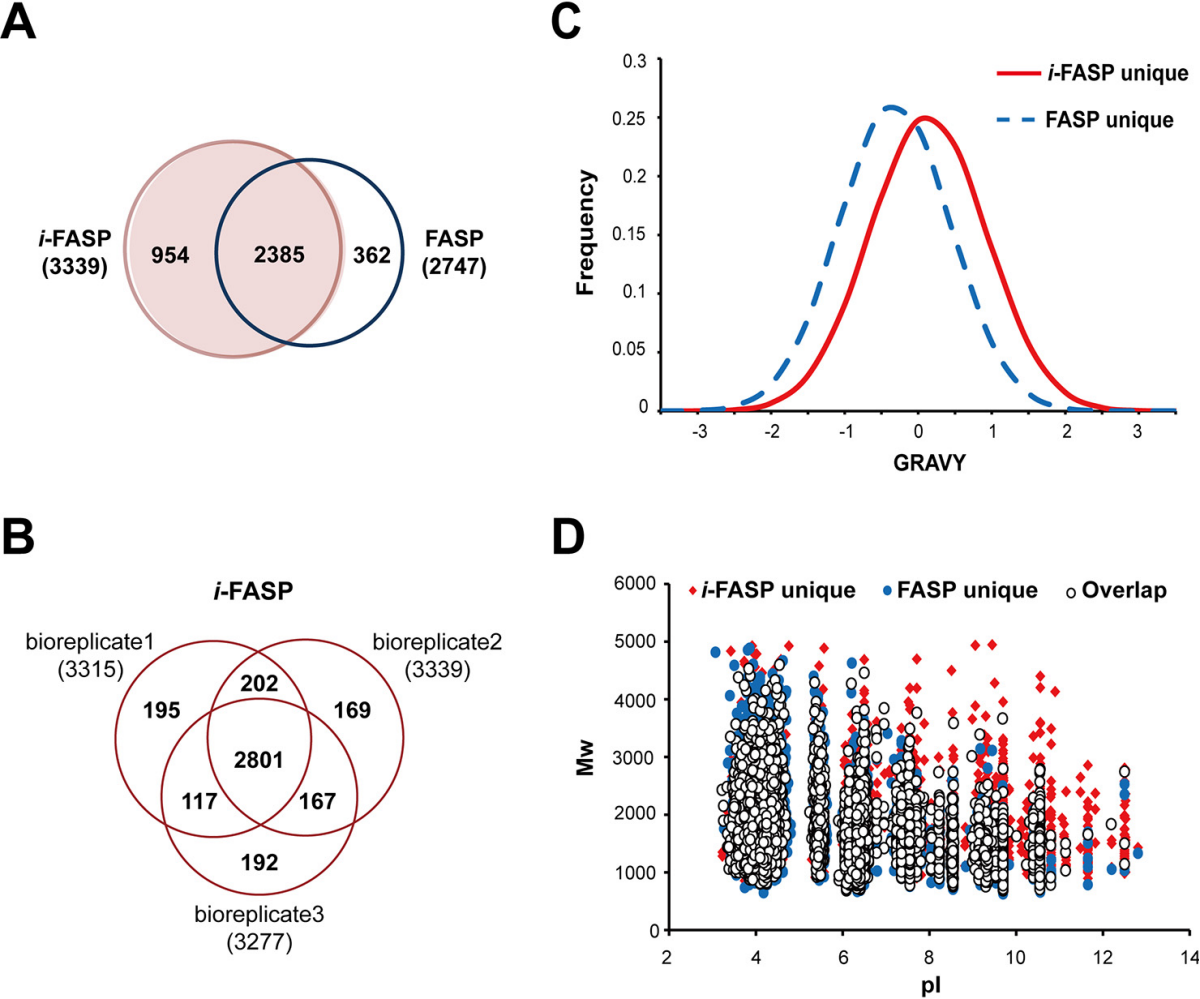
**Qualitative proteome analysis results with 10^6^ HeLa cells.***A*, Overlap of the proteins identified by the *i*-FASP and FASP methods, and *B*, overlap of the proteins identified from biological triplicates by the *i*-FASP method. The distribution of (*C*) the GRAVY values and (*D*) molecular weights (Mw) *versus* isoelectric points (pI) for the *i*-FASP-specific peptides (diamond), FASP-specific peptides (round) and shared peptides (circle).

Totally, the *i*-FASP method identified 3,339 proteins, providing 21.6% improvement compared with FASP method (Fig. 3*A* and supplemental Table S1), and 87% (2385/2747) of the proteins identified by FASP method were also detected with *i*-FASP method. Additionally, a high repeatability of 81.0% in biological triplicates was obtained by the *i*-FASP method, comparative to that obtained by FASP method of 79.6% (Fig. 3*B* and supplemental Fig. S3B). Impressively, the percentage of peptides containing missed cleavage sites identified from *i*-FASP method was 12.7 ± 0.3% (*n* = 3), twice less than that of FASP method (27.5 ± 0.8%). Afterward, the hydropathic character of the identified peptides was investigated. Compared with the FASP method, the distributions of peptide grand average of hydropathicity (GRAVY) values were noticeably shifted toward hydrophobicity for the solely identified peptides by using *i*-FASP method (shown in Fig. 3*C*). The scatter plots shown in Fig. 3*D* illustrated that many larger and more basic peptides were exclusively identified by *i*-FASP. There might be proton transfer reduction caused by the C12Im-Cl adduction, which would be further investigated in our future work.

Additionally, the protein recoveries of the *i*-FASP and FASP methods were measured by employing 25 µg of BSA as the standard protein, with the produced tryptic digests quantified by UV detection (214 nm) according to previous work (19). As a result, the recovery of FASP method was 66 ± 1% (*n* = 4), comparative to the published data as 50% (39), much lower than that of the *i*-FASP method as 90 ± 5% (*n* = 4). Additionally, with different starting sample amounts, the *i*-FASP method afforded recoveries ranging from 80% to 100% (supplemental Fig. S3C). It could be explained by that the residual C12Im-Cl favors the reduction of the nonspecific adsorption of proteins and peptides.

To further investigate the performance of the developed sample preparation method, equal amount of proteins extracted from HeLa cells with C12Im-Cl were treated with *i*-FASP and in-solution digestion methods, respectively. Thanks to the low sample loss attributed to the *in situ* processing, 35% improvement in protein identification was obtained by *i*-FASP method compared with in-solution digestion (supplemental Fig. S4A and supplemental Table S2), and 82% (1788/2186) of the proteins identified by in-solution method were also detected with *i*-FASP method. Compared with the in-solution method, a higher repeatability in biological triplicates was obtained by the *i*-FASP method (83.0% *versus* 78.6%). Expectedly, because of the impurities removal before protease digestion with the ultrafiltration unit, as few as 6.1% of the peptides identified with *i*-FASP method were predicted with missed cleavage sites, whereas only 68.3% of the peptides from in-solution digestion method were completely digested (supplemental Fig. S4B). Additionally, compared with high-abundance proteins (>100,000 copies per cell), much fewer low- (500–5000 copies per cell) and middle-abundance (5000–100,000 copies per cell) proteins were detected by in-solution method than *i*-FASP method (supplemental Fig. S4C).

Furthermore, the long-term compatibility with LC–MS/MS system by the residual C12Im-Cl from the *i*-FASP method was investigated. As shown in supplemental Fig. S5, after subjecting the digested peptides pretreated by *i*-FASP strategy into the same column for more than one month, there's almost no change for both peak intensity and peptide retention time. After running the samples for two months, the chromatographic peak intensity was almost as high as the 1^st^ run and the retention time shifted only 4-7 min for a 60 min LC gradient. Additionally, there was no significant peak broadening even after three months running (supplemental Fig. S5B). The above result showed a long-term stability of LC–MS system with the C12Im-Cl resided in *i*-FASP products.

The above results demonstrated that the *i*-FASP method allowed unbiased proteome analysis of both hydrophobic and hydrophilic proteins with high recovery contributed from three aspects. The first is the excellent solubilizing ability to hydrophobic proteins of C12Im-Cl at high concentration; the second is that with the introduction of ultrafiltration unit, most of the C12Im-Cl was depleted with NH_4_HCO_3_ washing, minimizing the sample loss caused by the harsh washing; the third is that because the small amount of C12Im-Cl was enzyme-friendly and MS-compatible, the residual extractant in the sample could help to enable the hydrophobic proteins remaining well spread over the surface of the spin column, ensuring their exposure to enzymatic digestion.

##### Qualitative Proteome Analysis of Trace Amount Cell Samples

**Fig. 4:**
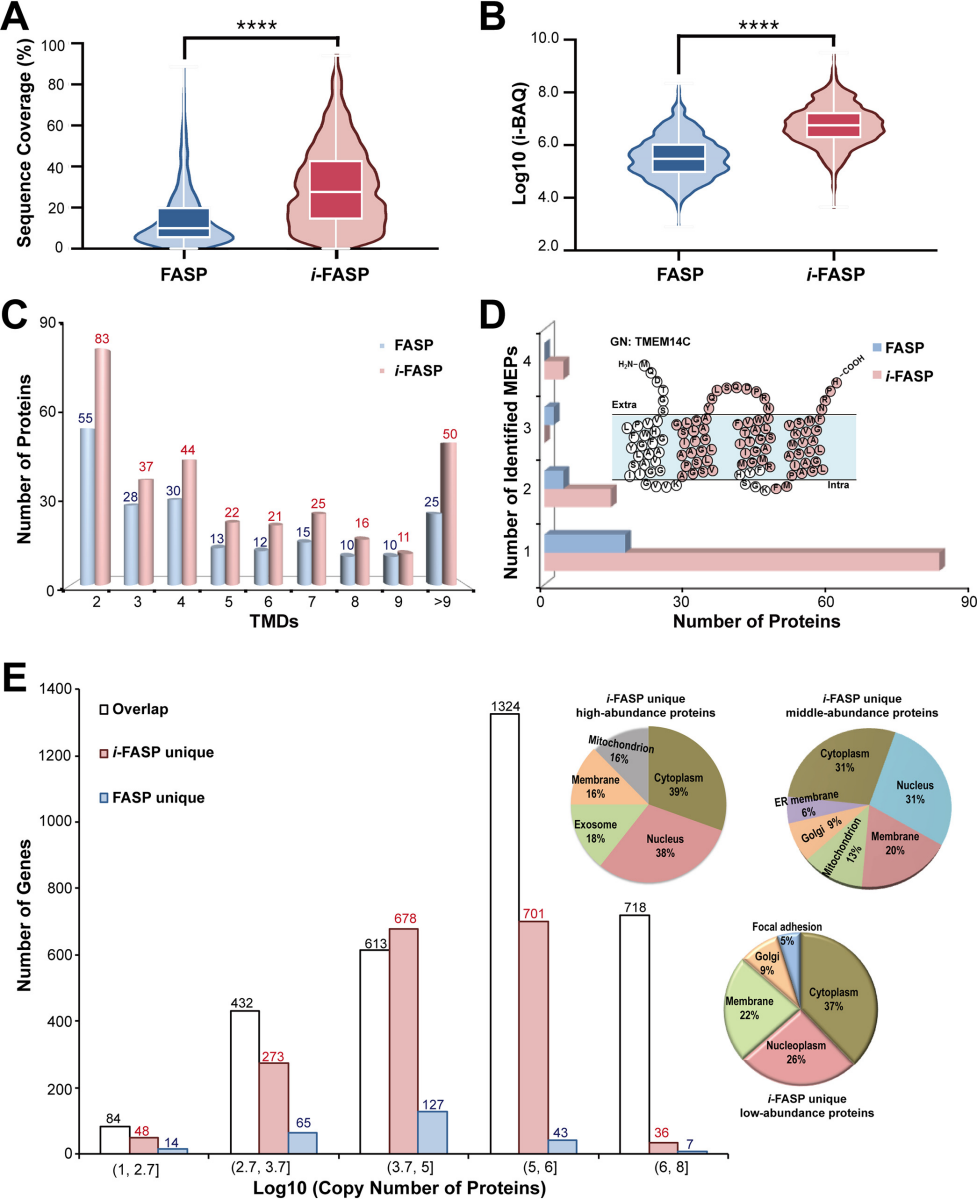
**Qualitative proteome analysis results for 10^3^ HeLa cells.** A comparison of (*A*) the abundance and (*B*) sequence coverage of commonly identified proteins from a 1000-cell sample prepared using the *i*-FASP and FASP methods; ****p < 0.0001 between *i*-FASP and FASP methods with two-tailed, Wilcoxon test. The distribution of (*C*) transmembrane domains (TMDs), (*D*) membrane-embedded peptides (MEPs) and (*E*) copy number of the proteins identified from the 1000-cell sample prepared using the *i*-FASP and FASP methods. Additionally, (*D*) the topology of the TMEM14C, including the four MEPs (*red* labeled amino acids) exclusively identified with *i*-FASP method, is shown in the inset. (*E*) The distribution of cellular component of the high-, middle- and low-abundance proteins uniquely identified with *i*-FASP method is shown in the inset.

Cell proteomics with relatively few cells is of great interest to the biological and clinical fields (40, 41). Because of tedious sample preparation, it is still difficult to complete deep coverage proteome analysis for trace amounts of samples. Based on its good performance, the *i*-FASP method was utilized to the analysis of proteins from 1000 HeLa cells. As shown in supplemental Table S3, 20,528 ± 226 peptides, corresponding to 3329 ± 41 proteins (*n* = 3) could be identified within 5 h of sample preparation and 1 h RPLC-MS/MS analysis, obviously improved compared with the number identified by FASP (10,613 ± 310 peptides, corresponding to 2173 ± 26 proteins, *n* = 3). The abundance of the proteins identified from 10^3^ HeLa cell sample prepared by *i*-FASP spanned ∼5.9 orders of magnitude, higher than that by FASP with 4.8 orders (supplemental Table S4). Additionally, nearly 90% (2410/2683) of the proteins identified with FASP method were covered by the *i*-FASP method. For those proteins both identified by the two methods, 93% of them with higher intensity (Fig. 4*A*) and sequence coverage (Fig. 4*B*) were achieved by *i*-FASP method. The distribution of the cellular components and biological functions for the identified proteins were investigated by using DAVID software. For the proteins identified with *i*-FASP method, 963 of them were assigned as membrane proteins, including 32.1% of them with more than one transmembrane domain (TMD) (supplemental Table S3). Although for the FASP method, only 741 of the identified proteins were assigned as membrane proteins, among which 26.7% were predicted to have ≥2 TMDs (Fig. 4*C*). Because of their high hydrophobicity, it is difficult to extract and identify the membrane embedded peptides (MEPs) by the FASP methods, whereas many more MEPs were efficiently identified by using *i*-FASP method (Fig. 4*D*). For example, transmembrane protein ^14^C (GN: TMEM14C), a 4-TM protein, could not be identified by FASP method considering its high percentage of MEPs, whereas the sequence coverage could be achieved as high as 67% by using *i*-FASP method (Fig. 4*D*).

Moreover, to evaluate the performance of *i*-FASP method to the low-abundance proteins, our results were referred to the copy numbers of proteins identified in previous work (4) (supplemental Table S5). As shown in Fig. 4*E*, most of the high-abundance proteins (>100,000 copies per cell), such as the cytoskeletal protein vimentin (gene name: VIM) with 20 million copies per cell (42), were successfully identified by both the *i*-FASP and FASP methods. However, for the middle- (5000–100,000 copies per cell) and low-abundance proteins (500-5,000 copies per cell), only few of them were uniquely identified with FASP method, whereas at least three times more proteins were uniquely identified by using *i*-FASP method, including the very low abundant proteins (<500 copies per cell). The cellular component of the proteins solely identified by *i*-FASP method was analyzed with DAVID software. Most of the low abundant proteins were assigned as cytoplasm, nucleus, plasma membrane, membrane and exosome proteins, whereas the top subcellular organelles for the high abundant proteins belonging to nucleus, cytoplasm, exosome and mitochondrion (Fig. 4*E* and supplemental Table S5). The above results demonstrated the superiority of *i*-FASP for pre-treating highly hydrophobic and low-abundance proteins, beneficial to improving the coverage of proteome analysis.

##### Quantitative Proteome Analysis of Human Hepatoma Tissue by Label-Free Method

The label-free quantitative method has been widely employed for the analysis of different samples with deep coverage and handy operation. However, it is extremely required the high reproducibility and protein identification sensitivity. Based on its excellent performance in qualitative proteome analysis, the *i*-FASP method was then employed to the label-free quantitative proteome analysis of human hepatoma tissues. In contrast to cell and other tissue samples, the high lipid content (5-10%) in human liver tissues can seriously affect the subsequent tryptic digestion and LC–MS/MS analysis, especially for the tumor tissues (43). In conventional lipid removal methods, the proteins extracted from the lipid-rich tissues were usually precipitated with acetone, resulting in serious sample loss (44). In our *i*-FASP method, different organic solvents, including methanol, ethanol, and isopropanol, mixed with equal volume of NH_4_HCO_3_, were respectively used as washing buffers to remove the lipids during C12Im-Cl removal process. And methanol mixed with equal volume of 50 mm NH_4_HCO_3_ was chosen as the optimal washing buffer based on the good performance of protein identification (supplemental Table S10). The cancer and para-carcinoma tissues of human liver samples were treated using *i*-FASP method. As a comparison, the same amounts of samples were back-to-back treated by using FASP method with 8 M urea as the extractant (45, 46). Overall, an average of 7,176 proteins (corresponded to 56,492 peptides) were identified from liver cancer tissue by *i*-FASP method, 6 and 16% increase compared with the protein and peptide number identified from FASP method (shown in supplemental Table S11 and supplemental Table S6). In addition, the higher Pearson's correlation coefficient between the replicate experiments was achieved by the *i*-FASP method, demonstrating the good reproducibility of this method (supplemental Fig. S6). These results can be attributed to the improved digestion efficiency and operational simplicity of the *i*-FASP method. The higher coverage and better repeatability obtained with the *i*-FASP method were prerequisites for achieving high accuracy and precision in the MS/MS-based label-free proteome quantitation method.

**Fig. 5:**
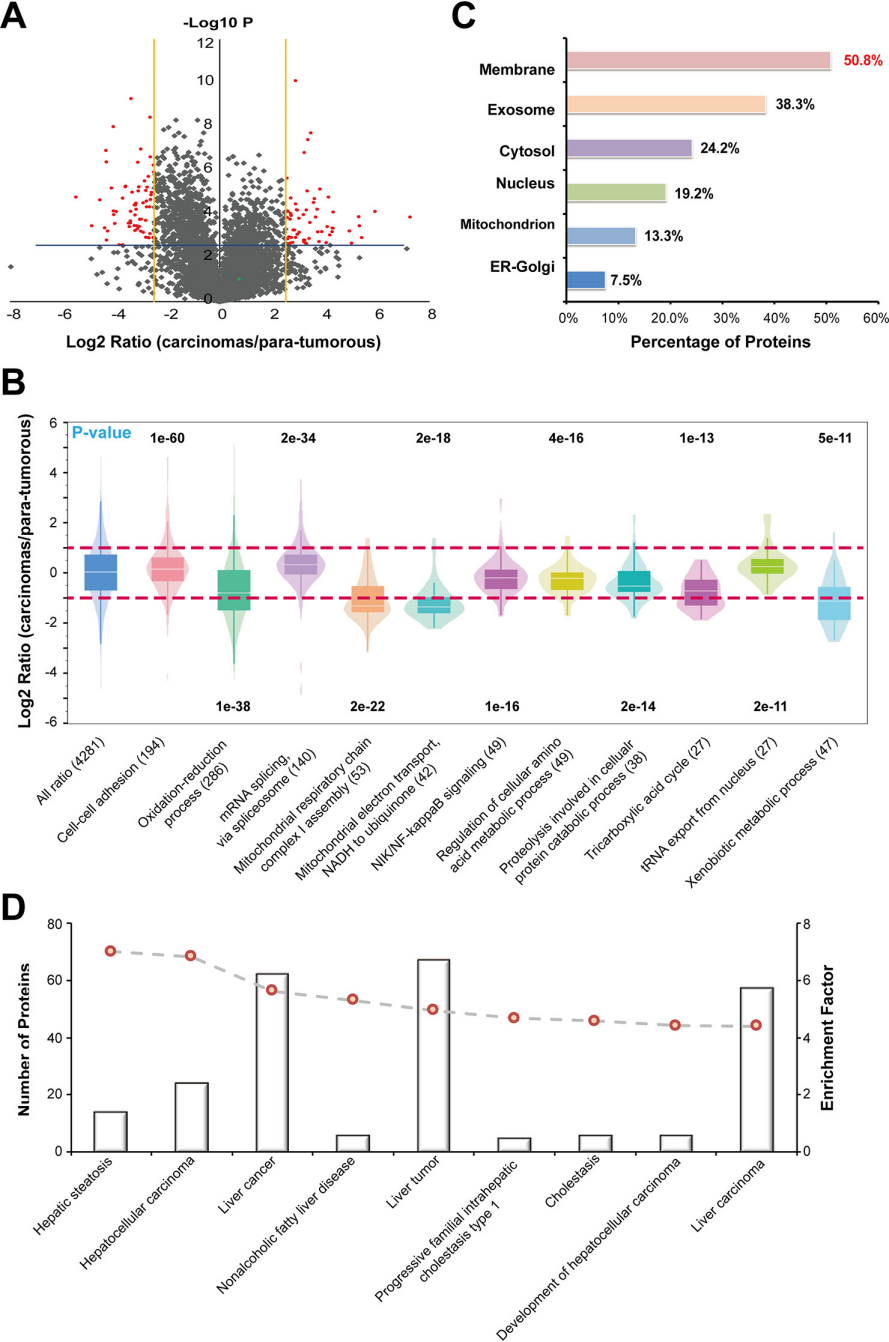
**Label-Free quantitative proteome analysis of human hepatoma tissue.***A*, Volcano plot and *B*, gene ontology analysis of the differentially expressed proteins quantified using the *i*-FASP method. *C,* Subcellular location of the 125 differentially expressed proteins identified by the *i*-FASP method. *D*, Number of molecules associated with diseases generated from Ingenuity Pathway Analysis database and our input data of 125 differentially expressed proteins.

The proteins quantified in all three replicates by the *i*-FASP and modified FASP methods were separately subjected to Benjamini-Hochberg (BH) FDR estimation, and those passed the 1% BH-FDR threshold were retained in the volcano plot (Fig. 5*A*). Totally, 4352 and 3946 proteins were confidently quantified with the *i*-FASP and m-FASP method, respectively (supplemental Table S7). To the best of our knowledge, this was the largest quantitative proteomic data set for single paired tumor and nontumor tissues of hepatocellular carcinoma. Gene ontology (GO) analysis suggests that whereas the proteins associated with biological processes such as cell-cell adhesion, mRNA splicing and tRNA export from nucleus are up-regulated, the proteins involved in the biological processes associated with mitochondrial functions such as oxidation-reduction process, assembly of respiratory chain complex I, electron transport and the tricarboxylic acid cycle are down-regulated (Fig. 5*B*). The difference between the functions regulated by the nucleus and mitochondria in cancer cells might be attributed to the mitochondria has its own genomic system independent from the nucleus, and they possess their own transcription, translation, and protein assembly machinery. Considering that the mitochondria electron transport system contributes to the metabolic remodeling in cancer cells by rebalancing the tumor bioenergetics toward glycolysis, the results of our GO analysis advances a possibility that the dysfunction in mitochondria electron transport might be partially responsible for the tumorigenesis of liver cancer.

With further control of the 2-fold standard deviation in the accuracy evaluation as the cutoff with significance, 125 and 58 proteins were confidently quantified as differentially expressed (DE) proteins by using the *i*-FASP and FASP method, respectively. Among them, 30 proteins were quantified with the consistent changes by both methods. We further explored the underlying information of the proteins exclusively quantified by two methods, and the other 28 DE proteins quantified using FASP method were all confidently identified by *i*-FASP method, but without showing significant changes caused by the poor repeatability in low-abundance proteins (supplemental Table S6). Expressively, among the DE proteins quantified by *i*-FASP method, the percentage of membrane proteins and low-abundance proteins, including extracellular exosomes and transcription factors, were as high as 50.8% and 40.8%, respectively (Fig. 5*C*). Additionally, among the proteins exclusively quantified with *i*-FASP method, 28 of them even could not be confidently identified when using the FASP method because of the limitation of this method in detecting proteins with low abundance and high hydrophobicity (supplemental Table S7).

The 125 DE proteins quantified by *i*-FASP were then subjected to disease network with Ingenuity Pathway Analysis (IPA) database, and most of the DE proteins were enriched to the liver-associated cancers (Fig. 5*D*). We further referred the biological functions of the DE proteins quantified by *i*-FASP to the previous reports (details shown in supplemental Table S7), and as many as 45.6% of the differential expressed proteins were reported to be associated with liver cancer, such as nestin (NES), and adenylyl cyclase-associated protein 1 (CAP1) (47, 48). Furthermore, 34.4% of the proteins were reported to be associated with other cancers, including macrophage-capping protein (CAPG), which is a as breast cancer biomarker (49), and perilipin-2 (PLIN2), which is a renal cell carcinoma biomarker (50). These proteins are potentially involved in the same oncogene pathway in liver cancer and may be potential biomarkers for multiple cancers. Interestingly, most of the DE proteins, not reported to be associated with tumors, were found closely related to cancer-associated proteins by using STRING software (supplemental Fig. S7A). The DE proteins were also subjected to the regulator effect analysis. The top two scoring networks were arteriosclerosis (supplemental Fig. S7B) and inflammation of liver (supplemental Fig. S5C). The predicted regulators, including C-C chemokine receptor (CCR2), peroxisome proliferator-activated receptor gamma coactivator (PPARGC1A), nuclear receptor (NR1I3) and peroxisomal acyl-CoA oxidase (ACOX1), might be biomarker candidates in liver cancer, but needs more biological validation in the future work.

## DISCUSSION

Sample preparation for proteome analysis typically involves the extraction of proteins from biological species and protease digestion. Therefore, the capacity for protein solubilization and protease compatibility of the extractant are critical elements for global proteome analysis. In this work, molecular dynamics simulation system was developed to play subsidiary role to experimental methods, facilitating to narrow down the scope of extractant candidate as well as reveal interior mechanism of extractants on both membrane proteins solubilization and trypsin biocompatibility at the molecular level. C12Im-Cl, a methylimidazolium-based ionic liquid, showed strong interactions with membrane proteins via its alkyl chain, whereas the conformation of trypsin, especially its catalytic triad, remained stable in this distinctive system.

Contributed by the merits of C12Im-Cl, an ionic liquid-based sample preparation method, *i*-FASP, was proposed for the in-depth coverage analysis of proteomics, especially for the low-abundance and highly hydrophobic proteins from trace amounts of samples. The *i*-FASP method showed superior protein extraction capacities and excellent compatibility with trypsin digestion, as well as high efficiency and throughput during sample preparation. When used in the qualitative analysis of cells and tissues, *i*-FASP showed great promise in deep-coverage proteome analysis. In combination with quantitative proteome strategies and the excellent performance of *i*-FASP in the analysis of membrane and low-abundance proteins, many differentially expressed proteins were confidently identified and quantified in our study with high accuracy and deep coverage. The results demonstrated the great potential of our *i*-FASP method to provide a rich resource for understanding the mechanisms of diseases and discovering novel biomarkers.

## DATA AVAILABILITY

The mass spectrometry proteomics data have been deposited to the ProteomeXchange. Consortium via the MassIVE partner repository with the dataset identifier MSV000083818.

National Key Research and Development Program of China (2017YFA0505003) to Qun Zhao, Baofeng Zhao, Lihua Zhang, and Yukui Zhang
National Key Research and Development Program of China (2016YFA0501401) to Qun Zhao, Baofeng Zhao, Lihua Zhang, and Yukui Zhang
NSFC | National Natural Science Foundation (21834006) to Qun Zhao, Baofeng Zhao, Lihua Zhang, and Yukui Zhang
NSFC | National Natural Science Foundation (91543201) to Qun Zhao, Baofeng Zhao, Lihua Zhang, and Yukui Zhang
NSFC | National Natural Science Foundation (21775150) to Qun Zhao, Baofeng Zhao, Lihua Zhang, and Yukui Zhang
CAS Key Project in Frontier Science (QYZDY-SSW-SLH017) to Qun Zhao, Baofeng Zhao, Lihua Zhang, and Yukui Zhang
Innovation program from DICP, CAS (DICP TMSR201601) to Qun Zhao, Baofeng Zhao, Lihua Zhang, and Yukui Zhang
Innovation program from DICP, CAS (DICP ZZBS201712) to Qun Zhao, Baofeng Zhao, Lihua Zhang, and Yukui Zhang
