# Supplementary material for: Molecular Dynamics Simulation-assisted Ionic Liquid Screening for Deep Coverage Proteome Analysis
Source: Mol Cell Proteomics. 2020 Nov 25;19(10):1724–37. doi: 10.1074/mcp.TIR119.001827 (PMC8015004; doi:10.1074/mcp.TIR119.001827)
Supplement: Supplementary file 1 [file mmc1.zip › 156541_3_supp_561943_qd6flv.pdf]

Supporting Information

Molecular Dynamics Simulation-assisted Ionic Liquid Screening for Deep Coverage Proteome Analysis

Fei Fang,[1,6,‡] Qun Zhao,[1,‡] Huiying Chu,[2,‡] Mingwei Liu,[3] Baofeng Zhao,[1] Zhen Liang,[1] Lihua Zhang,[1,*] Guohui Li,[2,*] Liming Wang,[4] Jun Qin,[3,5] and Yukui Zhang[1]

[1]CAS Key Laboratory of Separation Science for Analytical Chemistry, Dalian Institute of Chemical Physics, Chinese Academy of Science, National Chromatographic Research and Analysis Center, 457 Zhongshan Road, Dalian 116023, China.

[2]Laboratory of Molecular Modeling and Design, State Key Laboratory of Molecular Reaction Dynamics, Dalian Institute of Chemical Physics, Chinese Academy of Science, 457 Zhongshan Road, Dalian 116023, China

[3]State Key Laboratory of Proteomics, Beijing Proteome Research Center, Beijing Institute of Radiation Medicine; National Center for Protein Sciences (The PHOENIX Center, Beijing), Beijing 102206, China

[4]Division of Hepatobiliary and Pancreatic Surgery, Department of General Surgery, The Second Affiliated Hospital of Dalian Medical University, Dalian 116023, China

[5]Alkek Center for Molecular Discovery, Verna and Marrs McLean Department of Biochemistry and Molecular Biology, Department of Molecular and Cellular Biology, Baylor College of Medicine, Houston, Texas 77030, USA

[6]Current Affiliation: Department of Pharmacology and Chemical Biology, University of Pittsburgh, Pittsburgh, PA, 15261, USA.

* To whom correspondence should be addressed. E-mail: lihuazhang@dicp.ac.cn and ghli@dicp.ac.cn

‡ These authors contributed this work equally

List of contents:

- Experimental Procedures
- Supplemental Tables S1-S11
- Supplemental Figures S1-S7

Experimental Procedures

1.1 Recovery of *i*-FASP and FASP methods

The recovery of the whole sample preparation with *i*-FASP and FASP methods was evaluated using BSA as the sample. A set of 10, 15, 20, 25 and 30 μg of BSA were treated with urea-based sample preparation method (n=3), with the produced tryptic digests quantified by UV detection (214 nm) plotted as standard curve. With 25 μg of BSA respectively treated with *i*-FASP and FASP methods (n=4) and subjected to UV detection, the recovery of two methods was measure by referring the obtained UV absorbance value to the established standard curve.

To further investigate the recovery of *i*-FASP method, a set of 20, 45, 80, 115 and 140 μg of BSA were respectively treated with urea-based method (n=3), with the produced tryptic digests quantified by UV detection (214 nm) plotted as standard curve. With the tryptic digests respectively treated with *i*-FASP method from 20, 50, 75, 100 and 125 μg of BSA (n=3), the recoveries of *i*-FASP method with different amounts were measured.

1.2 Sample Preparation by *i*-FASP

Each aliquot containing 1,000 HeLa cells was solubilized by 15 μL of lysis buffer (10% C12Im-Cl and 0.1 M TCEP dissolved in 0.1 M Tris, pH 7.6, 1% protease inhibitor cocktail (v/v)). Subsequently, the sample was incubated at 95 °C for 3 min, and all the protein extract obtained was transferred to a 10k filter. The washing steps were the same as the procedures performed for the 10^6 cells, and the purified samples were subjected to proteolytic digestion by incubation with 100 ng of trypsin (dissolved in 50 μL of 10 mM NH_4HCO_3) at 37 °C for 3 h. The resulting solution was collected by centrifugation at 14,000×g for 10 min. Finally, the tryptic peptides were collected by centrifugation, and 50 μL of elution buffer (methanol mixed with equal volume of 10 mM NH_4HCO_3) was added to elute the peptide-rich solution twice.

The human liver cancer and para-carcinoma tissues were provided by the Second Affiliated Hospital of Dalian Medical University (Dalian, China), and the study was approved by the Institutional Review Board of the hospital. Informed consent was obtained from patients enrolled in this study. The para-carcinoma tissues were noncancerous tissues ≥2 cm away from the hepatic cancer nodules, whereas the cancer tissues were obtained from advanced stage hepatocellular carcinoma (HCC) patients and were removed surgically. The para-carcinoma tissues were verified by histopathological examination, and the invaded or microscopic metastatic cancer cells were excluded.

The liver tissues were lysed in an ice-cold homogenization buffer containing 10% C12Im-Cl, 100 mM Tris (pH 7.6), 1 mM EDTA, 0.5 mM EGTA, 1 mM PMSF, and 100 μL of protease inhibitor cocktail per 10 mL of homogenization buffer, and the protein concentration was determined by a BCA assay kit with BSA as the standard. Subsequently, the sample in 100 mM TCEP was reduced by incubating at 95 °C for 3 min. The cell debris was removed at 16,000×g for 5 min, and 150 μg of the clarified protein extract was transferred to a 10k filter. The washing steps for the tissue sample were the same as for the cells except that the washing buffer was replaced by the methanol mixed with equal volume of 50 mM NH_4HCO_3. Then, 100 μL of 10 mM NH_4HCO_3 containing modified trypsin (5 μg) was added, and the sample was digested overnight at 37°C. Finally, the tryptic peptides were collected by centrifugation, and 100 μL of water was added to elute the peptide-rich solution.

1.3 Sample Preparation by FASP

The samples were prepared using the FASP method following the procedures described previously with minor modifications (1). Each aliquot containing 1,000 HeLa cells was solubilized by 15 μL of lysis buffer (4% SDS, 0.1 M TCEP dissolved in 0.1 M Tris, pH 7.6, 1% protease inhibitor

cocktail (v/v)). Subsequently, the sample was incubated at 95 °C for 3 min, and the obtained protein extract was transferred to a 10k filter. The subsequent steps were the same as that of the preparation of the samples with 10^6 cells, and 100 ng of trypsin dissolved in 50 μL of 10 mM NH_4HCO_3 was added to the sample with incubation at 37 °C for 3 h. Finally, the tryptic peptides were collected by centrifugation, and 50 μL of water was added to elute the peptide-rich solution. The cancer and para-carcinoma tissues of human liver samples were separately lysed in ice-cold homogenization buffer containing 8 M urea, 100 mM Tris (pH 7.6), 1 mM EDTA, 0.5 mM EGTA, 1 mM PMSF and 100 μL of protease inhibitor cocktail per 10 mL of homogenization buffer, and the concentration of obtained proteins determined by using a BCA assay kit with BSA as a standard. Subsequently, proteins were reduced with 10 mM TCEP at 56 °C for 1 h, alkylated with 20 mM IAA in the dark at room temperature for 30 min, and transferred to a 10k filter. After centrifugation at 14,000×g at 20 °C for 15 min, proteins were retained, diluted with 200 μL of 50 mM NH_4HCO_3 and centrifuged at 14,000×g at 20 °C for 15 min. Subsequently, after the filter was washed twice with 200 μL of 50 mM NH_4HCO_3, 100 μL of 10 mM NH_4HCO_3 containing modified trypsin (5 μg) was added, and the sample was digested overnight at 37 °C. Finally, the tryptic peptides were collected by centrifugation, and 50 μL of water was added to elute the peptide-rich solution.

1.4 Nano RPLC-ESI-MS/MS Analysis

The separation of the tryptic peptides obtained from the HeLa cells and human liver tissue was performed on a C18 capillary column (15 cm, 75 μm i.d., 360 μm o.d.) packed with C18 silica particles (5 μm, 100 Å) under acidic conditions.

The separation of the tryptic peptides obtained from the sample containing 10^6 HeLa cells was performed on a C18 capillary column (15 cm, 75 μm i.d., 360 μm o.d.) packed with C18 silica

particles (5 μm, 100 Å) under acidic conditions. The two eluent buffers were H_2O with 2% ACN and 0.1% formic acid (FA) (A), and ACN with 2% H_2O and 0.1% FA (B), and both were at pH 3. The gradient of the mobile phase was set as follows: 5%-22% B in 65 min, 22%-35% B in 30 min, 30%-80% B in 5 min and maintained at 80% B for 10 min. The flow rate was 300 nL/min.

The peptides from the samples of 10^6 HeLa cells were analyzed using a Q-Exactive mass spectrometer equipped with a quaternary Surveyor pump and an ESI probe Ion Max Source with a microspray kit. The system was controlled by Xcalibur software version 2.1.0 from Thermo Fisher (Waltham, MA, USA) in the data-dependent acquisition mode. The capillary temperature was held at 250 ºC, and the mass spectrometer was operated in positive ion mode. Full MS scans were acquired in the orbitrap analyzer over the *m/z* 300–1,800 range with a resolution of 70,000 and the AGC target was 1e6. The 10 most intense ions were fragmented, and tandem mass spectra were acquired in the orbitrap mass analyzer with a mass resolution of 17,500 at *m/z* 200. The dynamic exclusion time was set to 40 s, and the maximum allowed ion accumulation times were 20 ms for MS scans and 60 ms for MS/MS scans.

Peptides (50 μg) from the cancer and para-carcinoma tissues of human liver samples were separated on a pipette-based reverse phase C18 column. The column was assembled following the StageTip principle (2) with minor modifications by stacking 3 mg Durashell RP resin (5 μm, 150 Å) on two layers of a 3 M Empore C18 Extraction Disk into a 100 μL micropipette tip. Peptides were loaded in mobile phase A (10 mM NH_4HCO_3, pH adjusted to 10 using $NH_3 \cdot H_2O$), and nine fractions were subsequently eluted with buffer solutions of 6%, 9%, 12%, 15%, 18%, 21%, 25%, 30% and 35% of mobile phases B (ACN, pH adjusted to 10 using $NH_3 \cdot H_2O$), respectively. These fractions were divided into the following three groups: the early eluting group (fractions 1-3), the middle eluting group (fractions 4-6) and the late eluting group (fractions 7-9). The, two fractions

from the early and late eluting groups with equal time intervals (i.e., fractions 1 and 7; fractions 2 and 8; and fractions 3 and 9) were mixed, and the obtained six fractions were vacuum dried and re-dissolved with 0.1% (v/v) FA in water for the second-dimension separation.

The separation of the six peptides fractions was performed on the same C18 capillary column mentioned above, but with different gradients of the mobile phases. The mobile phases in this instance were mixed by 97.9% H_2O with 2% ACN and 0.1% FA (A), and 79.9% ACN with 20% H2O and 0.1% FA (B). The separation of the six peptides fractions from liver tissues was performed on a C18 capillary column (15 cm, 75 μm i.d., 360 μm o.d.) packed with C18 silica particles (5 μm, 100 Å) under acidic conditions, but with different gradients of the mobile phase, mixed by 97.9% H_2O with 2% ACN and 0.1% formic acid (mobile phase A), and 79.9% ACN with 20% H_2O and 0.1% formic acid (mobile phase B). Fraction 1: 5%-12% B in 16 min, 12%-24% B in 35 min, 24%-32% B in 15 min, 32%-95% B in 1 min and maintained at 95% B for 8 min. Fraction 2: 6%-8% B in 7 min, 8%-15% B in 31 min, 15%-16% B in 2 min, 16%-32% B in 25 min, 32%-95% B in 1 min and maintained at 95% B for 9 min. Fraction 3: 8%-13% B in 15 min, 13%-16% B in 20 min, 16%-22% B in 26 min, 22%-35% B in 4 min, 35%-95% B in 1 min and maintained at 95% B for 9 min. Fraction 4: 8%-10% B in 5 min, 10%-14% B in 15 min, 14%-21% B in 35 min, 21%-35% B in 10 min, 35%-95% B in 1 min and maintained at 95% B for 9 min. Fraction 5: 5%-12% B in 1 min, 12%-18% B in 14 min, 18%-25% B in 47 min, 25%-35% B in 3 min, 35%-95% B in 1 min and maintained at 95% B for 9 min. Fraction 6: 5%-15% B in 1 min, 15%-20% B in 20 min, 20%-28% B in 39 min, 28%-35% B in 5 min, 35%-95% B in 1 min and maintained at 95% B for 9 min.

For 1,000 HeLa cells, the gradient of the mobile phase was set as follows: 5%-10% B in 16 min, 10%-22% B in 35 min, 22%-30% B in 15 min, 30%-95% in 1 min and maintained at 95% B for 8

min. The flow rate was 600 nL/min.

The identification of proteins in 1,000 HeLa cells and liver tissues was carried out on an Orbitrap Fusion Lumos Tribrid mass spectrometer equipped with an EASY-nLC™ 1200 and an ESI probe Ion Max NG™ Ion Source with a microspray kit. The system was controlled by Xcalibur software version 4.0 from Thermo Fisher in the data-dependent acquisition mode. The mass spectrometer was operated in positive ion mode. Full MS scans were acquired in the orbitrap analyzer over the *m/z* 300-1,400 range with a resolution of 120,000 and the AGC target was 1e6. Peptides (charge states from 2+ to 6+) were selected by the quadrupole. The maximum allowed ion accumulation times were 50 ms for MS scans and 35 ms for MS/MS scans.

REFERENCES

1. Wisniewski, J. R., Zougman, A., Nagaraj, N., and Mann, M. (2009) Universal sample preparation method for proteome analysis. *Nat Methods* 6, 359-362

2. Rappsilber, J., Mann, M., and Ishihama, Y. (2007) Protocol for micro-purification, enrichment, pre-fractionation and storage of peptides for proteomics using StageTips. *Nat Protoc* 2, 1896-1906

Supplemental Tables

Table S1. Interaction energies between the bacteriorhodopsin (BR) and various ionic liquids with different cation groups $[A]^+$ and chlorine $[Cl]^-$ as methyltributylammonium chloride (MTBA-Cl), methyltributylphosphonium chloride (MTBP-Cl), 1-dodecyl-3-methylimidazolium chloride (C12Im-Cl) and 1-dodecyl-3-methylpyridinium chloride (C12Py-Cl) were calculated, respectively.

**Ionic liquids (1%, w/v)**	**Electrostatic interaction force ($[A]^+$-BR) (KJ/mol)**	**Van der Waals force ($[A]^+$-BR) (KJ/mol)**	**Electrostatic interaction force ($[Cl]^-$-BR) (KJ/mol)**	**Van der Waals force ($[Cl]^-$-BR) (KJ/mol)**	**$E_{ele+vdW}$ (KJ/mol)**
**MTBA-Cl**	-19.84	-683.69	-31.44	0.64	-734.33
**MTBP-Cl**	-33.23	-470.22	-34.48	0.07	-537.86
**C12Im-Cl**	-74.771	-744.038	-45.275	-0.159	-864.243
**C12Py-Cl**	-134.49	-687.45	-34.8	-0.11	-856.85

Table S2. Interaction energies between the bacteriorhodopsin (BR) and methylimidazolium chloride with different length of alkyl side chains (n = 2, 4, 6, 8, 10, 12, 14, 16) (CnIm-Cl) were calculated.

Ionic liquids (1%, w/v)	Electrostatic interaction force ($[CnIm]^+$-BR) (KJ/mol)	Van der Waals force ($[CnIm]^+$-BR) (KJ/mol)	Electrostatic interaction force ($[Cl]^-$-BR) (KJ/mol)	Van der Waals force($[Cl]^-$-BR) (KJ/mol)	$E_{ele+vdW}$ (KJ/mol)
**C2Im-Cl**	-77.494	-120.396	-44.645	0.858	-241.677
**C4Im-Cl**	-152.828	-198.604	-70.548	2.540	-419.440
**C6Im-Cl**	-110.496	-462.413	-51.430	0.474	-623.865
**C8Im-Cl**	-128.022	-590.698	-70.042	0.624	-788.138
**C10Im-Cl**	-89.843	-682.222	-27.077	-1.086	-800.228
**C12Im-Cl**	-74.771	-744.038	-45.275	-0.159	-864.243
**C14Im-Cl**	-77.548	-742.824	-43.071	1.0457	-862.397
**C16Im-Cl**	-77.570	-756.755	-29.324	0.559	-863.090

Table S3. Interaction energies between the bacteriorhodopsin (BR) and methylimidazolium bromide with different length of alkyl side chains (n = 2, 4, 6, 8, 10, 12, 14) (CnIm-Br) were calculated.

**Ionic liquids (1%, w/v)**	**Electrostatic interaction force ($[CnIm]^+$-BR) (KJ/mol)**	**Van der Waals force ($[CnIm]^+$-BR) (KJ/mol)**	**Electrostatic interaction force (Br^--BR) (KJ/mol)**	**Van der Waals force (Br^--BR) (KJ/mol)**	**$E_{ele+vdW}$ (KJ/mol)**
**C2Im-Br**	-88.63	-104.97	-51.75	-0.9	-246.25
**C4Im-Br**	-135.63	-252.87	-62.44	0.96	-449.98
**C6Im-Br**	-113.98	-511.52	-42.71	0.71	-667.5
**C8Im-Br**	-110.19	-613.445	-7.98	-1.96	-733.575
**C10Im-Br**	-74.79	-721.4	-45.82	0.09	-841.92
**C12Im-Br**	-91.42	-847.46	-13.72	-0.31	-952.91
**C14Im-Br**	-108.74	-670.1	-8.25	-0.66	-787.75

Table S4. Interaction energies between the bacteriorhodopsin (BR) and methylimidazolium tetrafluoroborate with different length of alkyl side chains (n = 2, 4, 6, 8, 10, 12, 14) (CnIm-BF_4) were calculated.

**Ionic liquids (1%, w/v)**	**Electrostatic interaction force ($[CnIm]^+$-BR) (KJ/mol)**	**Van der Waals force ($[CnIm]^+$-BR) (KJ/mol)**	**Electrostatic interaction force (BF_4^--BR) (KJ/mol)**	**Van der Waals force (BF_4^--BR) (KJ/mol)**	**$E_{ele+vdW}$ (KJ/mol)**
**C2Im-BF_4**	-45.72	-91.13	-75.87	-14.15	-226.87
**C4Im-BF_4**	-118.15	-206.9	-30.66	-16.6	-372.31
**C6Im-BF_4**	-65.72	-432.97	-105.9	-23.83	-628.42
**C8Im-BF_4**	-126.569	-543.78	-65.42	-13.54	-749.309
**C10Im-BF_4**	-78.56	-721.37	-46.93	-12.72	-859.58
**C12Im-BF_4**	-97.76	-908.18	-77.9	-13.11	-1096.95
**C14Im-BF_4**	-89.15	-583.496	-28.46	-8.25	-709.356

Table S5. Interaction energies between the bacteriorhodopsin (BR) and methylimidazolium chloride with different length of alkyl side chains (n = 2, 6, 8, 10, 12) and -CH=CH_2 substitute (CH2=CH-CnIm-Cl) were calculated.

**Ionic liquids (1%, w/v)**	**Electrostatic interaction force ([CH_2=CH-CnIm]$^+$-BR) (KJ/mol)**	**Van der Waals force ([CH_2=CH-CnIm]$^+$-BR) (KJ/mol)**	**Electrostatic interaction force ([Cl]$^-$-BR) (KJ/mol)**	**Van der Waals force ([Cl]$^-$-BR) (KJ/mol)**	**$E_{ele+vdW}$ (KJ/mol)**
**CH_2=CH-C2Im-Cl**	-92.62	-172.57	-51.68	0.07	-316.8
**CH_2=CH-C6Im-Cl**	-101.89	-584.57	-50.88	0.22	-737.12
**CH_2=CH-C8Im-Cl**	-86.14	-673.47	-56.4	0.69	-815.32
**CH_2=CH-C10Im-Cl**	-100.93	-941.44	-38.01	-0.23	-1080.61
**CH_2=CH-C12Im-Cl**	-38.12	-571.29	-20.35	0.2	-629.56

Table S6. Interaction energies between the bacteriorhodopsin (BR) and methylimidazolium chloride with different length of alkyl side chains (n = 4, 8, 10, 12, 14) and -CN substitute (CN-CnIm-Cl) were calculated.

Ionic liquids (1%, w/v)	Electrostatic interaction force ($[CN\text{-}CnIm]^+$-BR) (KJ/mol)	Van der Waals force ($[CN\text{-}CnIm]^+$-BR) (KJ/mol)	Electrostatic interaction force ($[Cl]^-$-BR) (KJ/mol)	Van der Waals force ($[Cl]^-$-BR) (KJ/mol)	$E_{ele+vdW}$ (KJ/mol)
**CN-C4Im-Cl**	-59.6	-76.97	-50.18	0.39	-186.36
**CN-C8Im-Cl**	-96.9	-296.32	-36.97	0	-430.19
**CN-C10Im-Cl**	-111.34	-515.39	-38.88	0.04	-665.57
**CN-C12Im-Cl**	-139.6	-860.27	-27.34	-0.62	-1027.83
**CN-C14Im-Cl**	-144.08	-622.68	-17.18	0	-783.94

Table S7. Interaction energies between the bacteriorhodopsin (BR) and methylimidazolium chloride with different length of alkyl side chains (n = 4, 8, 10) and -OH substitute (OH-CnIm-Cl) were calculated.

**Ionic liquids (1%, w/v)**	**Electrostatic interaction force ($[OH\text{-}CnIm]^{+}$-BR) (KJ/mol)**	**Van der Waals force ($[OH\text{-}CnIm]^{+}$-BR) (KJ/mol)**	**Electrostatic interaction force ($[Cl]^{-}$-BR) (KJ/mol)**	**Van der Waals force ($[Cl]^{-}$-BR) (KJ/mol)**	**$E_{ele+vdW}$ (KJ/mol)**
**OH-C4Im-Cl**	-124.71	-95.7	-51.56	0.24	-271.73
**OH-C8Im-Cl**	-137.62	-329.94	-40.17	0.13	-507.6
**OH-C10Im-Cl**	-169.21	-534.53	-40.84	0.24	-744.34

Table S8. Interaction energies between the bacteriorhodopsin (BR) and methylimidazolium chloride with different length of alkyl side chains (n = 4, 8, 10, 12) and $-NH_2$ substitute (NH_2-CnIm-Cl) were calculated.

**Ionic liquids (1%, w/v)**	**Electrostatic interaction force ($[NH_2$-CnIm$]^+$-BR) (KJ/mol)**	**Van der Waals force ($[NH_2$-CnIm$]^+$-BR) (KJ/mol)**	**Electrostatic interaction force ($[Cl]^-$-BR) (KJ/mol)**	**Van der Waals force ($[Cl]^-$-BR) (KJ/mol)**	**$E_{ele+vdW}$ (KJ/mol)**
**NH_2-C4Im-Cl**	-95.83	-276.93	-53.26	0.04	-425.98
**NH_2-C8Im-Cl**	-125.93	-748.74	-39.91	-0.91	-915.49
**NH_2-C10Im-Cl**	-95.89	-790.31	-68.35	0.8	-953.75
**NH_2-C12Im-Cl**	-115.59	-897.44	-17.33	-0.43	-1030.79

Table S9. Interaction energies between the bacteriorhodopsin (BR) and three different ionic liquids, 1-dodecyl-3-methylimidazolium chloride (C12Im-Cl) and 1-butyl-3-methylimidazolium thiocyanate (C4Im-SCN).

**Ionic liquids (1%, w/v)**	**Electrostatic interaction force ($[A]^+$-BR) (KJ/mol)**	**Van der Waals force ($[A]^+$-BR) (KJ/mol)**	**Electrostatic interaction force ($[Cl]^-$-BR) (KJ/mol)**	**Van der Waals force ($[Cl]^-$-BR) (KJ/mol)**	**$E_{ele+vdW}$ (KJ/mol)**
**C12Im-Cl**	-74.771	-744.038	-45.275	-0.159	-864.243
**C4Im-SCN**	-111.523	-163.021	-62.4156	-10.0007	-346.96

Table S10. Summary of protein groups, peptides, and integral membrane proteins (IMPs) identified for each triplicate tissue sample that were prepared using either NH_4HCO_3 buffer or 50% methanol (v/v) as washing buffer.

	NH_4HCO_3 buffer washing			50% methanol washing		
	**T1**	**T2**	**T3**	**T1**	**T2**	**T3**
**Proteins**	2434±15	2416±27	2338±8	2666±8	2676±13	2556±9
**Peptides**	12447±42	12278±98	12013±36	14613±120	14383±185	13642±110
**IMPs**	424±2	417±2	400±3	481±1	479±5	454±6

Table S11. Quantified number of protein groups, peptides and spectral utilization of human liver cancer and para-carcinoma tissues with sample prepared by *i*-FASP and modified FASP (m-FASP) methods.

Sample	Methods	Protein	Peptide	Spectral Utilization (%)
**Cancer tissue**	***i*-FASP**	7176 ± 38	56492 ± 419	32.1 ± 0.2
	**m-FASP**	6750 ± 54	48627 ± 439	26.6 ± 0.4
**Para-carcinoma tissue**	***i*-FASP**	5734 ± 25	42469 ± 115	30.2 ± 0.4
	**m-FASP**	5378 ± 73	35698 ± 561	25.8 ± 0.8

Supplemental Figures

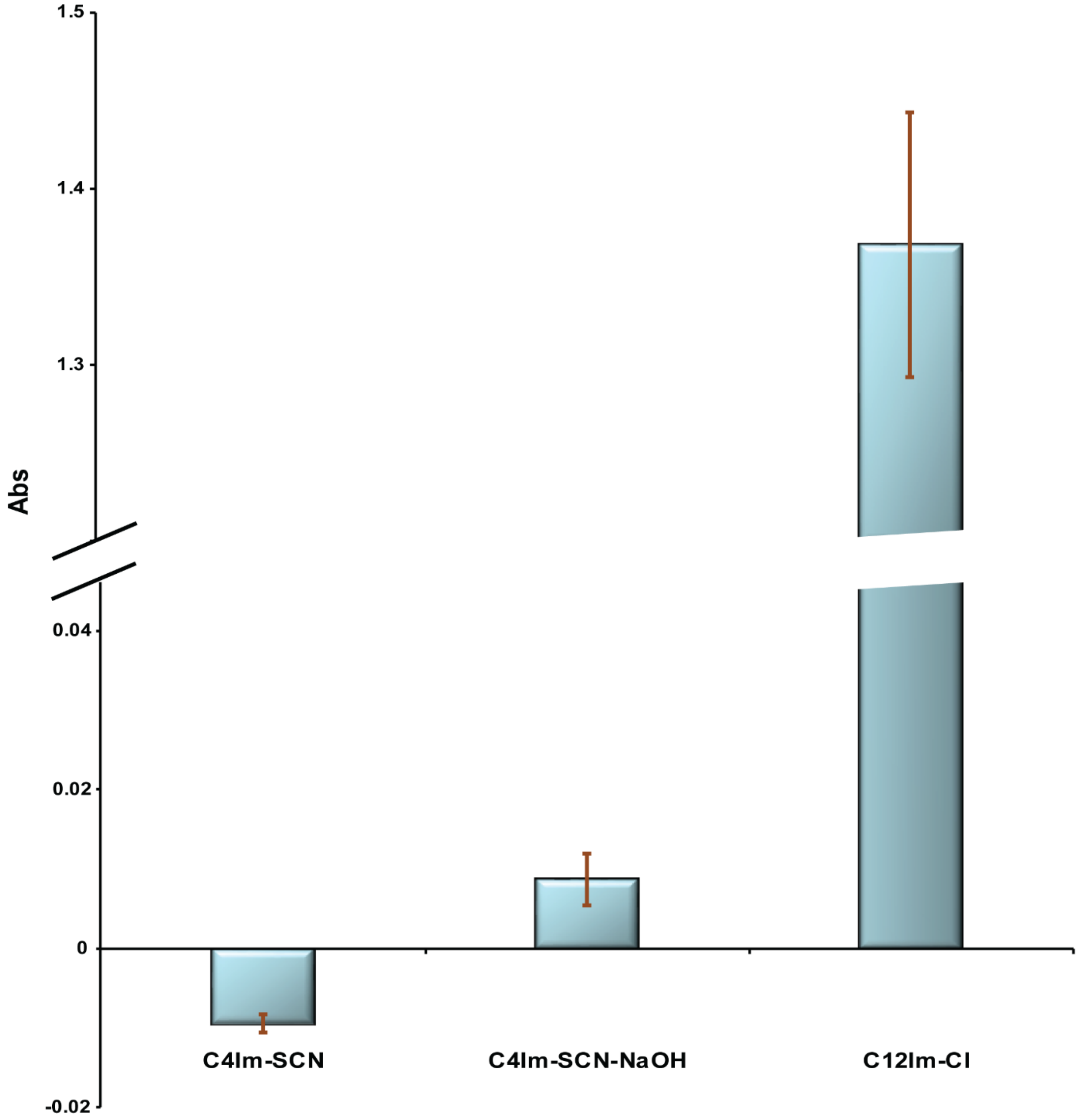

Figure S1. Comparison on the relative solubility of bacteriorhodopsin in various ILs. Equal aliquots of bacteriorhodopsin (100 μg) were respectively solubilized in 25 μL of each IL at 1% (m/v), including C4Im-SCN in 50 mM NH_4HCO_3 buffer, C4Im-SCN mixed with 0.5 M NaOH, as well as C12Im-Cl in 50 mM NH_4HCO_3 buffer.

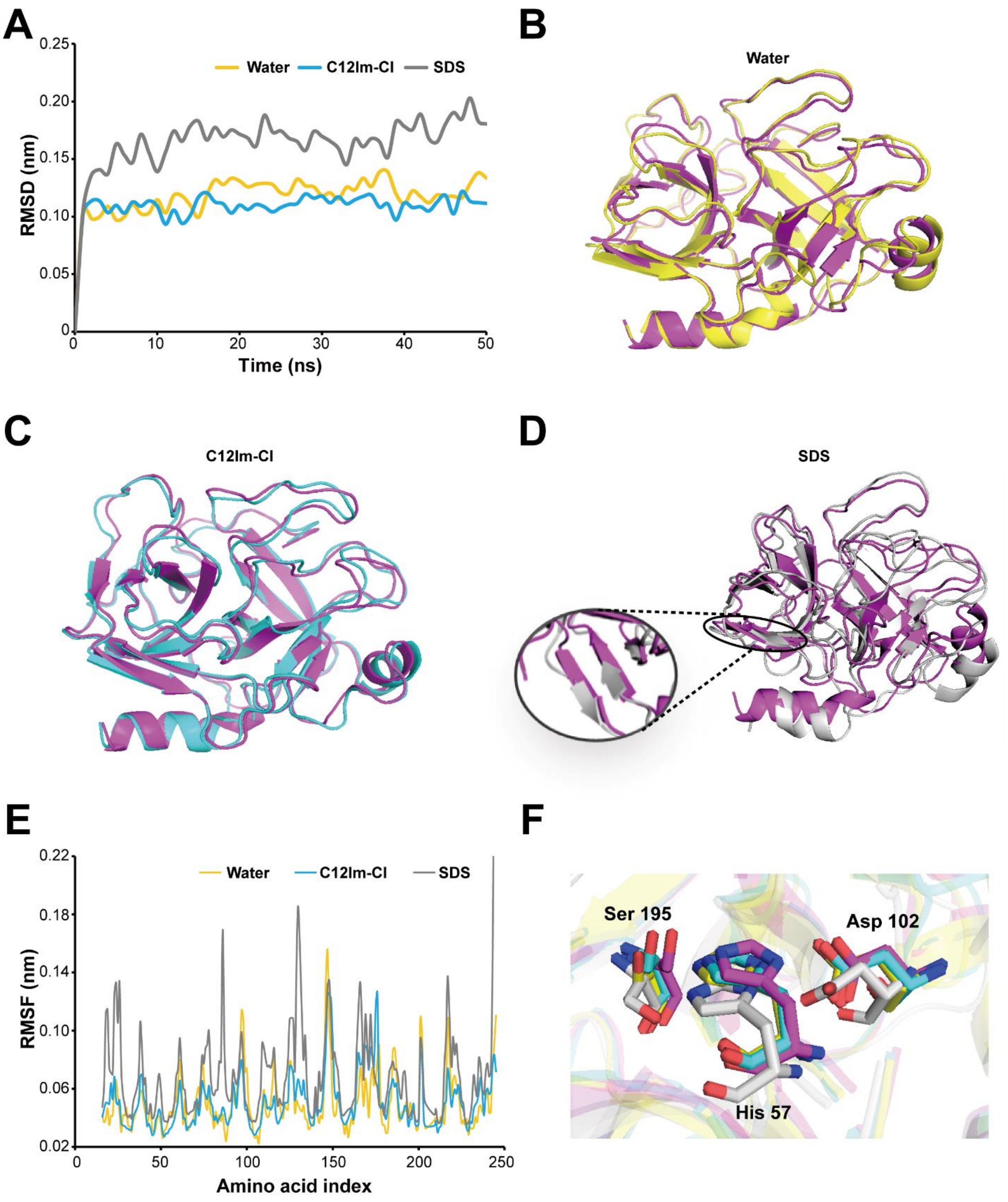

Figure S2. Compatibility of solvents with trypsin evaluated by molecular dynamics simulation. (A) RMSD of trypsin in different solvents relative to the native crystal structure. The conformation of trypsin in (B) water, (C) C12Im-Cl, and (D) SDS compared with the native crystal

conformation (PDB code 4AN7). (E) root-mean-square fluctuation (RMSF) values of each amino acid of trypsin dissolved in water, C12Im-Cl and SDS systems. and (F) the conformational changes of the catalytic triad in the four systems. The native crystal of trypsin is shown in magenta, trypsin in water is shown in yellow, trypsin in C12Im-Cl is shown in cyan, and trypsin in SDS is shown in grey.

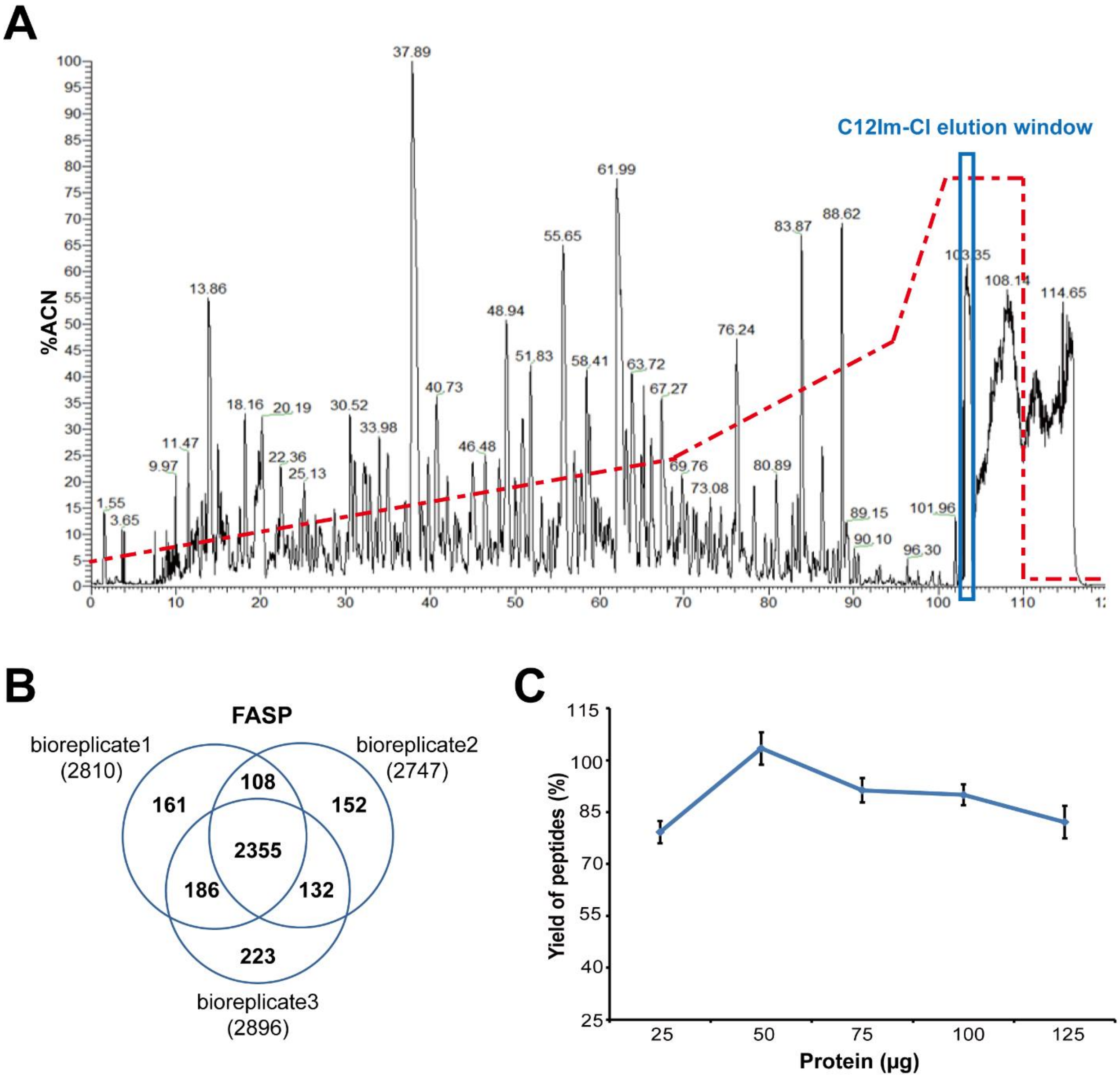

Figure S3. Establishment of *i*-FASP method. (A) Base peak chromatograph of the HeLa cell digest treated by *i*-FASP method, (B) overlap of the proteins identified from biological triplicates by the FASP method, and (C) the recovery of BSA digest with different amounts treated by *i*-FASP method.

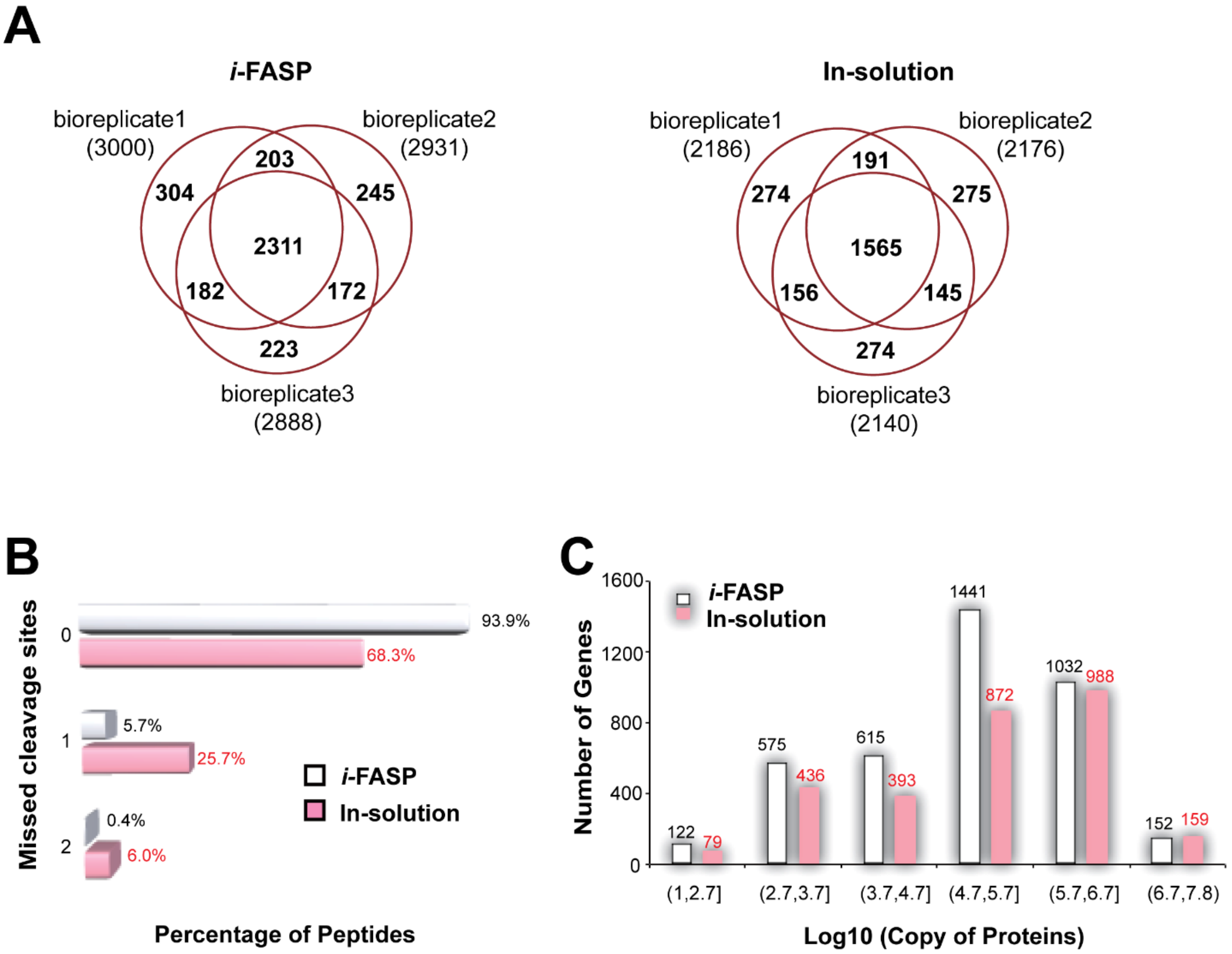

Figure S4. Qualitative proteome analysis results with 10^6 of HeLa cells pretreated by the *i*-FASP and in-solution methods. (A) Overlap of the proteins identified from biological triplicates by the *i*-FASP and in-solution methods. The distribution of (B) peptides with missed cleavage sites and (C) copy number of the proteins identified from the sample prepared using the *i*-FASP and in-solution methods.

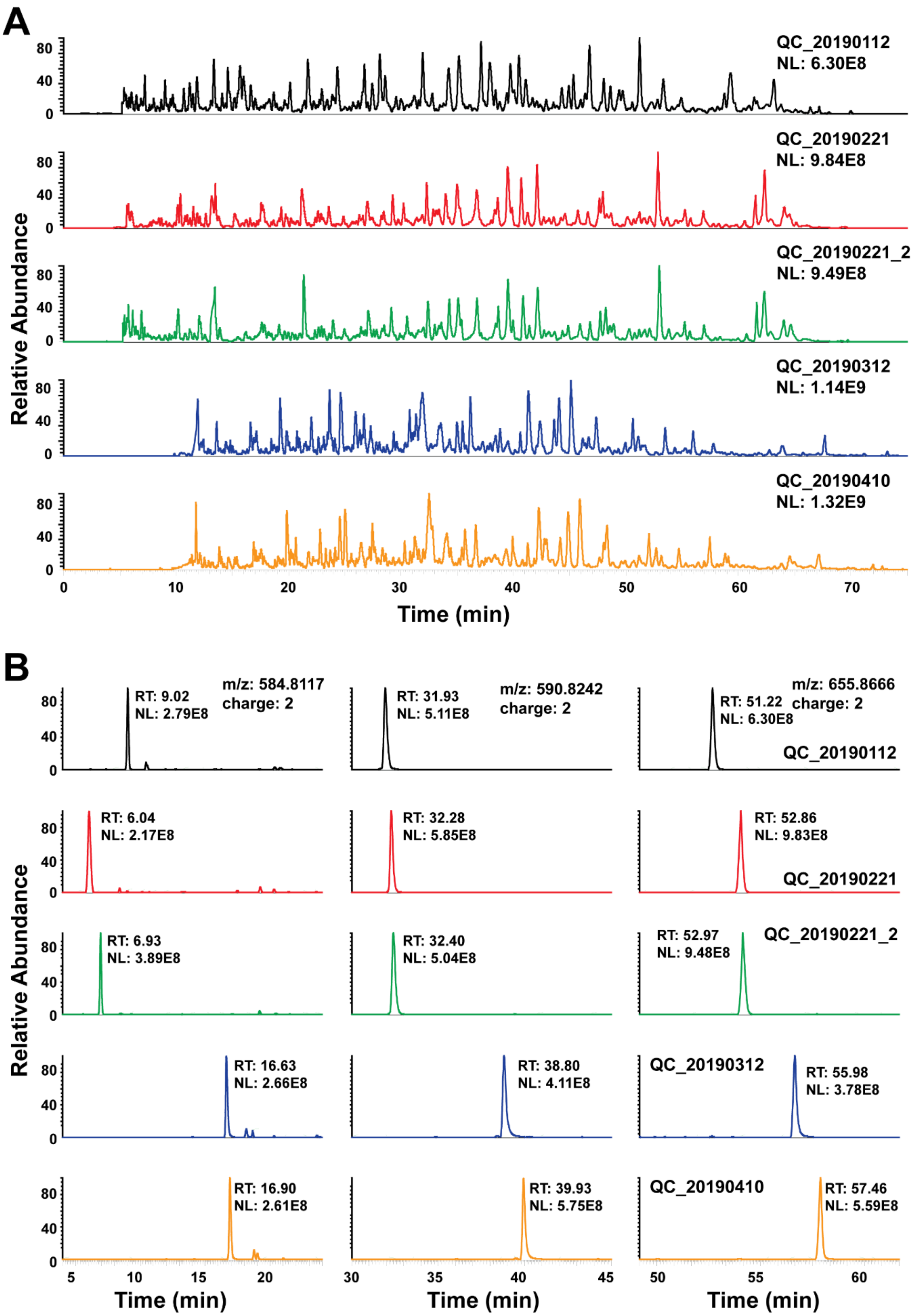
A
QC_20190112
NL: 6.30E8
QC_20190221
NL: 9.84E8
QC_20190221_2
NL: 9.49E8
QC_20190312
NL: 1.14E9
QC_20190410
NL: 1.32E9
Relative Abundance
Time (min)
B
m/z: 584.8117
charge: 2
m/z: 590.8242
charge: 2
m/z: 655.8666
charge: 2
RT: 9.02
NL: 2.79E8
RT: 31.93
NL: 5.11E8
RT: 51.22
NL: 6.30E8
QC_20190112
RT: 6.04
NL: 2.17E8
RT: 32.28
NL: 5.85E8
RT: 52.86
NL: 9.83E8
QC_20190221
RT: 6.93
NL: 3.89E8
RT: 32.40
NL: 5.04E8
RT: 52.97
NL: 9.48E8
QC_20190221_2
RT: 16.63
NL: 2.66E8
RT: 38.80
NL: 4.11E8
QC_20190312
RT: 55.98
NL: 3.78E8
RT: 16.90
NL: 2.61E8
RT: 39.93
NL: 5.75E8
QC_20190410
RT: 57.46
NL: 5.59E8
Relative Abundance
Time (min)

Figure S5. Long-term stability of LC-MS system with injection of samples pretreated by *i*-FASP method. (A) Base peak chromatograph and (B) extracted peaks of the HeLa cell digest prepared by *i*-FASP method with same separation column within three months.

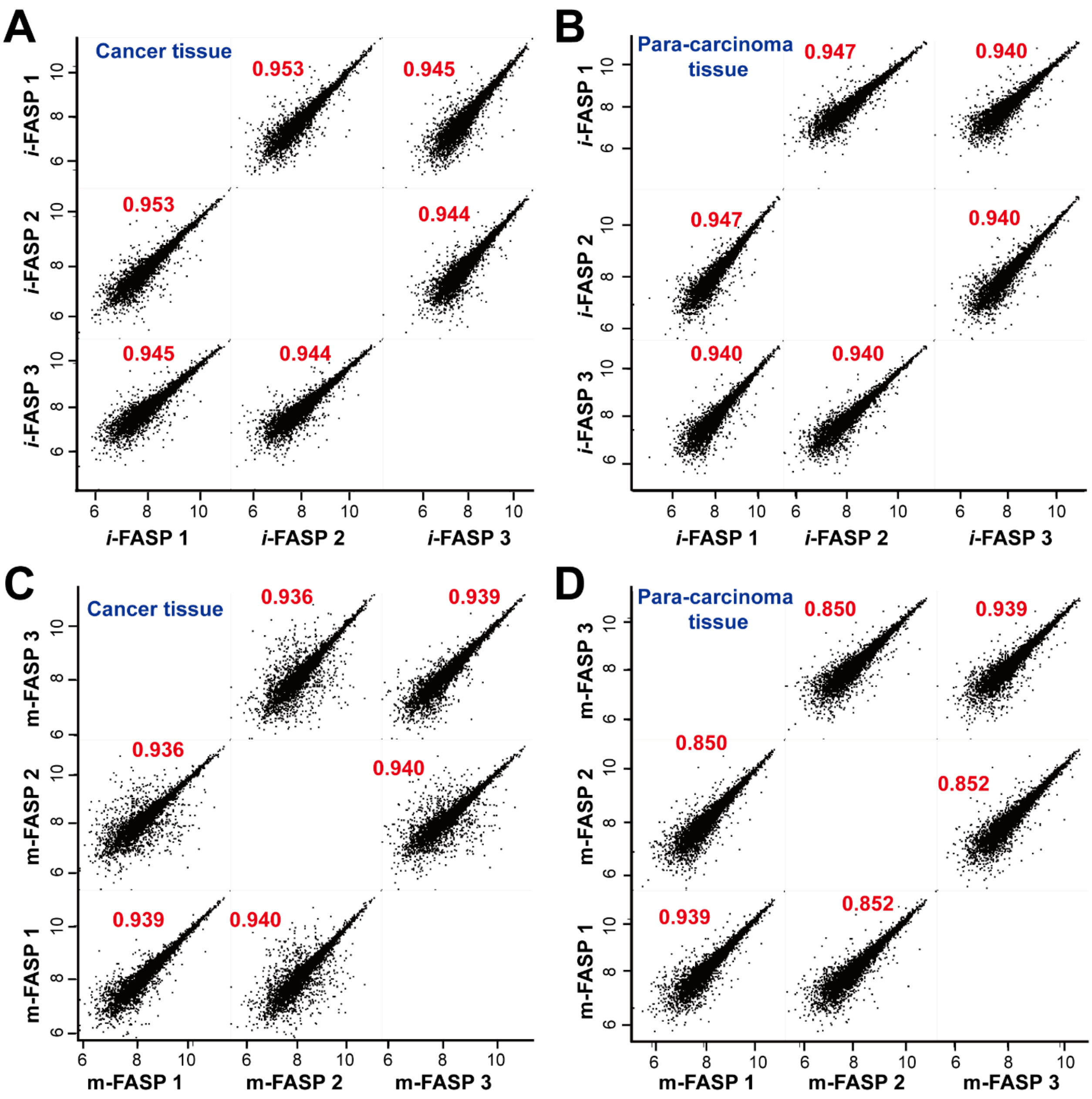

Figure S6. Repeatability of label-free quantitative analysis with samples prepared by *i*-FASP and m-FASP methods. Correlations between *i*-FASP method for (A) cancer and (B) para-carcinoma tissue preparation, and protein quantification with m-FASP method for (C) cancer and (D) para-carcinoma tissue preparation were shown.

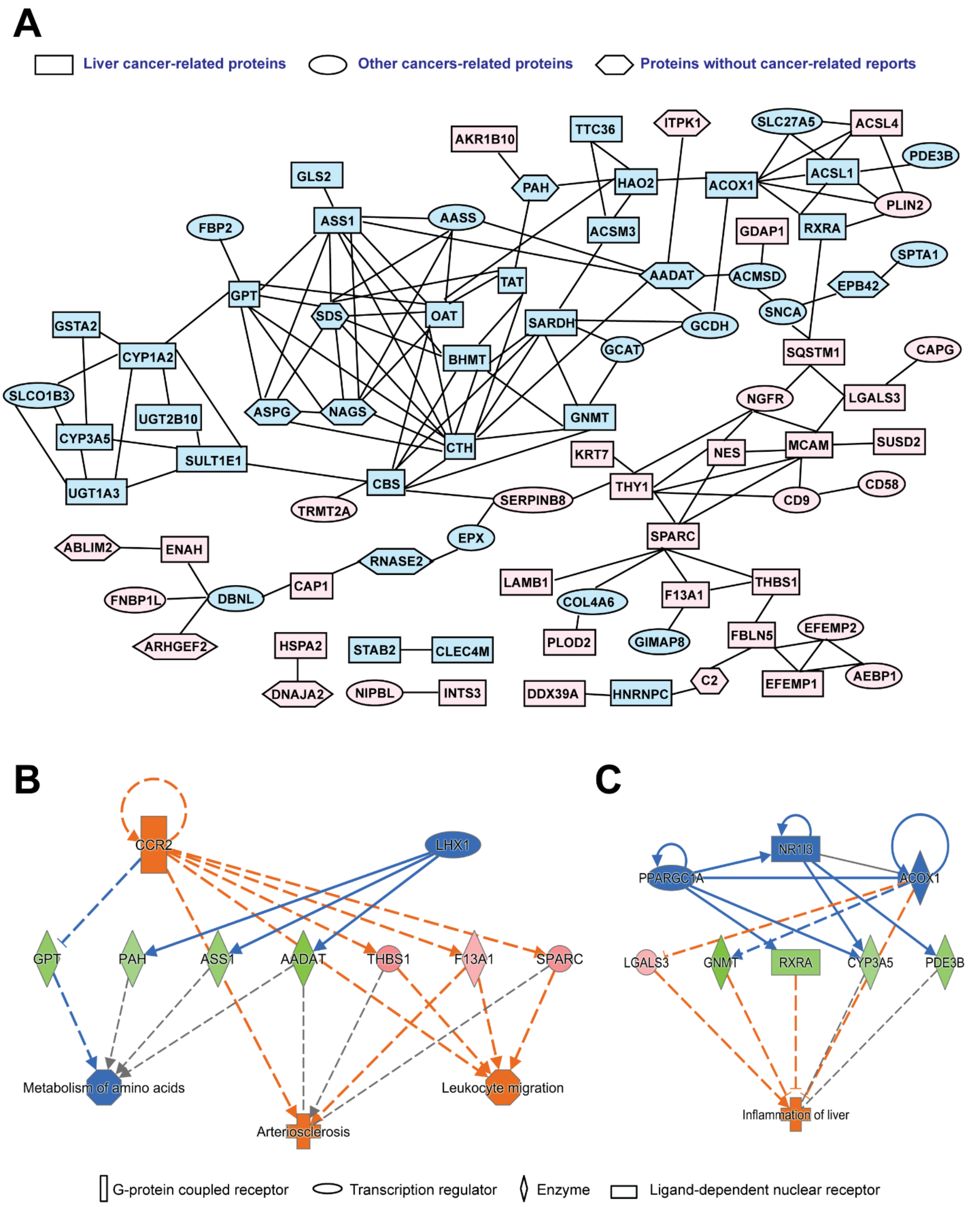

Figure S7. The network of protein interactions of the 125 differentially expressed proteins quantified by the *i*-FASP method. (A) The network of protein interactions obtained from STRING

database. The up-regulated and down-regulated proteins for cancer in comparison with para-carcinoma tissue were marked by red and blue, respectively. The top two scoring network obtained from the regulator effects analysis in Ingenuity Pathway Analysis system, including (B) arteriosclerosis, leukocyte migration and metabolism of amino acids and (C) inflammation of liver. Orange and blue nodes/lines represented activation and inhibition, respectively; red and green represented up-regulated and down-regulated proteins for cancer in comparison with para-carcinoma tissue. Solid arrows indicate a positive effect while dotted arrows indicate a negative effect.
