# Supplementary material for: Molecular Dynamics Simulation-assisted Ionic Liquid Screening for Deep Coverage Proteome Analysis
Source: Mol Cell Proteomics. 2020 Nov 25;19(10):1724–37. doi: 10.1074/mcp.TIR119.001827 (PMC8015004; doi:10.1074/mcp.TIR119.001827)
Supplement: Supplementary file 1 [file mmc1.zip › 156541_3_supp_561946_qd6flv.pdf]

Supporting Information

Recovery of *i*-FASP and FASP methods; Sample Preparation by *i*-FASP; Sample Preparation by FASP; Nano RPLC-ESI-MS/MS Analysis

Comparison of the relative solubility of bacteriorhodopsin in various ILs (Figure S1)

Compatibility of solvents with trypsin evaluated by molecular dynamics simulation (Figure S2)

Establishment of *i*-FASP method (Figure S3)

Qualitative proteome analysis results with 10^6 of HeLa cells (Figure S4)

Long-term stability of LC-MS system with rejection of samples treated by *i*-FASP method (Figure S5)

Repeatability of label-free quantitative analysis with samples prepared by *i*-FASP and m-FASP methods, respectively (Figure S6)

The network of protein interactions of the 125 differential proteins identified by the *i*-FASP method (Figure S7)

Interaction energies between the bacteriorhodopsin (BR) and various ionic liquids with different cation groups $[A]^+$ and chlorine $[Cl]^-$ as methyltributylammonium chloride (MTBA-Cl), methyltributylphosphonium chloride (MTBP-Cl), 1-dodecyl-3-methylimidazolium chloride (C12Im-Cl) and 1-dodecyl-3-methylpyridinium chloride (C12Py-Cl) were calculated, respectively (Table S1)

Interaction energies between the bacteriorhodopsin (BR) and methylimidazolium chloride with different length of alkyl side chains (n = 2, 4, 6, 8, 10, 12, 14, 16) (CnIm-Cl) were calculated (Table S2)

Interaction energies between the bacteriorhodopsin (BR) and methylimidazolium bromide with different length of alkyl side chains (n = 2, 4, 6, 8, 10, 12, 14) (CnIm-Br) were calculated (Table S3)

Interaction energies between the bacteriorhodopsin (BR) and methylimidazolium tetrafluoroborate with different length of alkyl side chains (n = 2, 4, 6, 8, 10, 12, 14) (CnIm-BF_4) were calculated (Table S4)

Interaction energies between the bacteriorhodopsin (BR) and methylimidazolium chloride with different length of alkyl side chains (n = 2, 6, 8, 10, 12) and -CH=CH_2 substitute (CH2=CH-CnIm-Cl) were calculated (Table S5)

Interaction energies between the bacteriorhodopsin (BR) and methylimidazolium chloride with different length of alkyl side chains (n = 4, 8, 10, 12, 14) and -CN substitute (CN-CnIm-Cl) were calculated (Table S6)

Interaction energies between the bacteriorhodopsin (BR) and methylimidazolium chloride with different length of alkyl side chains (n = 4, 8, 10) and -OH substitute (OH-CnIm-Cl) were calculated (Table S7)

Interaction energies between the bacteriorhodopsin (BR) and methylimidazolium chloride with different length of alkyl side chains (n = 4, 8, 10, 12) and -NH_2 substitute (NH_2-CnIm-Cl) were calculated (Table S8)

Interaction energies between the bacteriorhodopsin (BR) and three different ionic liquids, 1-dodecyl-3-methylimidazolium chloride (C12Im-Cl) and 1-butyl-3-methylimidazolium thiocyanate (C4Im-SCN) (Table S9)

Summary of protein groups, peptides, and integral membrane proteins (IMPs) identified for each triplicate tissue sample (Table S10)

Quantified number of protein groups, peptides and spectral utilization of human liver cancer and para-carcinoma tissues with sample prepared by *i*-FASP and modified FASP methods (Table S11)

Qualitative proteome analysis of 1×10^6 HeLa cells with *i*-FASP and FASP methods (Supplementary Table 1) (XLSX)

Qualitative proteome analysis of 1×10^6 HeLa cells with *i*-FASP and In-solution digestion methods (Supplementary Table 2) (XLSX)

Qualitative proteome analysis, distribution of TMDs and cellular components of the 1000-cell proteome with *i*-FASP and FASP methods (Supplementary Table 3) (XLSX)

Abundance and sequence coverage of each protein identified from 1×10^3 HeLa cells with *i*-FASP and FASP methods (Supplementary Table 4) (XLSX)

Distribution of copy number, cellular components and biological functions for proteins identified from 1×10^3 HeLa cells with *i*-FASP and FASP methods (Supplementary Table 5) (XLSX)

Qualitative proteome analysis from human liver cancer and para-tumorous tissues with *i*-FASP and m-FASP methods (Supplementary Table 6) (XLSX)

Distribution of ratio and biological process for the proteins quantified from human liver cancer and para-tumorous tissues with *i*-FASP method; differentially expressed proteins quantified from

human liver cancer and para-tumorous tissues with *i*-FASP and m-FASP methods (Supplementary Table 7) (XLSX)
